# Determinants of *mer* Promoter Activity from *Pseudomonas aeruginosa*

**DOI:** 10.3390/genes15040490

**Published:** 2024-04-13

**Authors:** Qingyuan Hu, Jue Wang, Chunhong Liu, Yu Feng, Hao Chen

**Affiliations:** State Key Laboratory of Coordination Chemistry, School of Chemistry and Chemical Engineering, Nanjing University, Nanjing 210023, China; huqingyuan1991@163.com (Q.H.); wj690189386@163.com (J.W.); liuchunhong1111@126.com (C.L.); 602023240027@smail.nju.edu.cn (Y.F.)

**Keywords:** *mer* promoter, expression activity, spacer length, position −13, guanine, synergistic regulation

## Abstract

Since the MerR family is known for its special regulatory mechanism, we aimed to explore which factors determine the expression activity of the *mer* promoter. The *Tn501/Tn21 mer* promoter contains an abnormally long spacer (19 bp) between the −35 and −10 elements, which is essential for the unique DNA distortion mechanism. To further understand the role of base sequences in the *mer* promoter spacer, this study systematically engineered a series of mutant derivatives and used luminescent and fluorescent reporter genes to investigate the expression activity of these derivatives. The results reveal that the expression activity of the *mer* promoter is synergistically modulated by the spacer length (17 bp is optimal) and the region upstream of −10 (especially −13G). The spacing is regulated by MerR transcription factors through symmetrical sequences, and −13G presumably functions through interaction with the RNA polymerase sigma-70 subunit.

## 1. Introduction

Human activities constantly accelerate the emission of heavy metals into the environment [1,2,3], increasingly threatening the well-being of organisms and the ecosystem [4,5,6]. According to the World Health Organization, mercury generally has the lowest maximum allowable value (MAV) among heavy metals due to its significant toxicity and bioaccumulation [7,8]. To cope with the toxicity of mercury from natural sources [9,10,11], prokaryotes have evolved various mechanisms, including the well-known system of mercury resistance (*mer*) genes regulated by the transcription factor (TF) MerR [12,13,14,15].

The best-studied mercury resistance *mer* operons were first characterized in the transposons *Tn501* from *Pseudomonas aeruginosa* and *Tn21* from the *Shigella flexneri* R100 plasmid (*Tn501/Tn21*) [16,17]. The *Tn501/Tn21 mer* promoter (*P_mer_*) contains a spacer that is abnormally longer (19 bp) than the optimal spacer between the −35 and −10 elements [18,19,20,21], which is important for the MerR regulatory system. Previous genetic studies performed base pair mutations in the *mer* operon [22,23,24], including several single-base-pair deletions (−30C, −24T, −18C, and −15/−14A) and a double-base-pair deletion (^−14^AG^−13^) in the spacer [25,26,27]. Deletions of 1–2 bp in the spacer resulted in stronger constitutive promoters, providing support for the unique DNA distortion mechanism of the TF MerR to activate transcription [28,29,30]. In the absence of mercury ions, MerR is a repressor of *P_mer_*. Upon binding to mercury ions, MerR distorts promoter DNA to shorten the spacing between the −35 and −10 elements for RNA polymerase recognition and activates transcription [31,32,33,34,35]. Although no complex crystals of MerR, Hg(II), or DNA from Gram-negative bacteria have been resolved, TF-induced DNA distortion was observed in complex crystals of other MerR family TFs with similar structures [36,37,38,39,40,41,42,43,44].

Transcription is activated by inducing promoter DNA kinking and undertwisting, resulting in phasing and spacing parameters close to a 17 bp promoter [45,46,47]. In addition, some studies support an optimal spacer length of 17 bp [48,49,50], while others suggest an optimal spacer length of 16–18 bp [51,52,53]. Typically, even if the spacer differs from the optimum by only 1 bp, the promoter activity is significantly reduced [54,55,56,57,58]. However, a *P_mer_* derivative with a 17 bp spacer exhibited constitutive activity similar to that of derivatives with 18 bp spacers in previous in vivo studies [27]. Therefore, we became interested in what causes this phenomenon and whether the function of the base sequences in the spacer is simply for DNA distortion.

In this work, we systematically engineered mutations in four regions (−30 to −28, −25 to −23, −20 to −18, and −15 to −13) in the spacer between the −35 and −10 elements of *P_mer_* (from −31 to −13) to investigate the expression activity of the derivatives. Expression levels were analyzed semi-quantitatively by using the sensitive bioluminescent reporter genes *luxAB* or the visible fluorescent reporter gene *sfGFP* instead of the *mer* genes [59,60]. The results indicate that the spacing (regulated through symmetrical sequence) and the region upstream of −10 (−15 to −13, especially −13G) synergistically regulate *P_mer_* activity, contributing to the further understanding of the function of the spacer sequence between the −35 and −10 elements of *P_mer_*.

## 2. Materials and Methods

### 2.1. Strains, Genes, Plasmids, and Primers

The *Escherichia coli* strains were DH5α (*supE44* Δ*lac*U169(*φ*80lacZΔM15) *hsdR17 recA1 endA1 thi-1 gyrA96 relA1*) and BL21(DE3) (*F– ompT hsdS_B_ (r_B_–*, *m_B_–) gal dcm (DE3)*). DH5α was used for cloning, luminescence assays, and fluorescence assays. BL21(DE3) was used for MerR protein expression. The *merR* gene and *mer* operator–promoter gene (*merOP*) from *P. aeruginosa*, *sfGFP* gene, promoters *P_cpcBA_* [61,62], *P_cpc560_* [63], *P_psbAII_* [64], *P_rnpB_* [65], *P_tic_* [66], and *P_cmv_* [67] were synthesized by GenScript (GenScript Biotech Co., Ltd., Nanjing, Jiangsu, China) (Table S1). The *luxA* and *luxB* genes were PCR-amplified from the pBBR-lux plasmid [59]. The pBBR-lux plasmid was kindly provided by Prof. Jun Zhu from Nanjing Agricultural University. Plasmids were as follows: pET28a, pBBR-lux, pMPmerL, pMPmerS, pPmerL, pPmerS, pLPmerS, pTf16, pPcpc560M, pPcpcBAS, pPcpc560S, pPticS, PpsbAIIS, pPrnpBS, and pPcmvS (see below). The primers were synthesized by Sangon Biotech (Sangon Biotech (Shanghai) Co., Ltd., Shanghai, China) (Tables S2 and S3).

### 2.2. Designing Base Pair Deletion Derivatives of the mer Promoter

**Figure 1:**
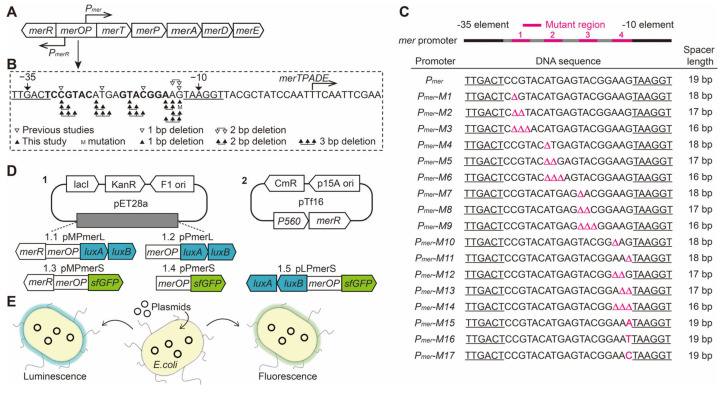
Schematic diagram of the expression activity assay for derivatives of the *mer* promoter. (**A**) Structure of the *mer* operon. (**B**) Sequence of the *merOP* region and position of mutations in the *mer* promoter spacer region. Hollow triangles represent reported mutants and solid triangles represent mutants in this paper. (**C**) Wild-type and derivative M1–M17 promoter sequences and spacer lengths. Mutation sites are labeled in pink. The −35 and −10 elements are marked with an underscore. Mutant regions are marked in pink. (**D**) Plasmid maps of the vectors constructed in this study. pMPmerL, pPmerL, pMPmerS, pPmerS, pLPmerS, and pPcpc560M. (**E**) Visible and quantitative detection of promoter expression activity in *E. coli* by luminescence or fluorescence assays.

The *mer* operon studied in this work is from the *P. aeruginosa* genome (identical to the *Tn501/Tn21 mer* promoter) and includes *merR*, *merOP*, *merT*, *merP*, *merA*, *merD,* and *merE* (Figure 1A), where *merOP* refers to the intergenic region harboring the divergent and partially overlapping *merR* promoter (*P_merR_*) and *merT* promoter (*P_mer_*). The anti-mercury (*mer*) system encoded by the *mer* operon consists of inner membrane cytoplasmic protein (MerT), periplasmic mercury(II) scavenging protein (MerP), mercury ion reductase (MerA), potential co-regulator (MerD), and transmembrane protein (MerE) [14,31,68].

Base pair deletions were designed more comprehensively in this work, ranging from 1 to 3 bp and located in the four regions (−30 to −28, −25 to −23, −20 to −18, and −15 to −13) (Figure 1B,C and Figure S1). These positions are regularly spaced at 2 bp intervals. The −30 to −28 and −20 to −18 regions are in the symmetrical sequence (^−32^TCCGTACXXXXGTACGGA^−15^), the −25 to −23 region is between the symmetrical sequences, and the −15 to −13 region is upstream of the −10 element. The spacer length becomes 18–16 bp by deleting 1–3 bp in the spacer (Figure S2).

### 2.3. DNA Manipulations and Mutagenesis

QuickCut™ series restriction endonucleases, T4 DNA ligase, and PrimeSTAR HS DNA Polymerase were purchased from Takara (Takara Biomedical Technology (Beijing) Co., Ltd., Beijing, China), and 2× Taq Master Mix (Dye Plus) and Phanta^®^ Max Super-Fidelity DNA Polymerase were purchased from Vazyme (Nanjing Vazyme Biotech Co., Ltd., Nanjing, Jiangsu, China). They were used according to the manufacturer’s recommendations.

The *merR-merOP* genes and *luxAB* (or *sfGFP*) genes were amplified by PCR using Phanta^®^ Max Super-Fidelity DNA Polymerase. PCR amplification procedure: (1) pre-denaturation: 95 °C for 3 min; (2) cycle: 95 °C, 15 s; 54 °C, 15 s; 72 °C, 75 s, 34 cycle; (3) extension: 72 °C, 5 min; (4) preservation: 4 °C. The final concentrations of components: (1) Phanta Max buffer: 1×; (2) dNTP Mix: 0.2 mM each; (3) primer: 0.4 μM each; (4) template DNA: 20 pg/μL; (5) Phanta Max Super-Fidelity DNA polymerase: 1 U/50 μL. The final volume of the PCR is 50 μL. The *merR-merOP* genes and *luxAB* (or *sfGFP*) genes were spliced by overlapping using Phanta^®^ Max Super-Fidelity DNA Polymerase. The spliced fragments were integrated into plasmid pET28a by restriction endonuclease sites *Xba*I and *Eco*RI using restriction endonucleases and T4 DNA ligase to form vector pMPmerL (or pMPmerS) (Figure 1D and Figure S3). The vectors pPmerL and pPmerS were constructed from pMPmerL and pMPmerS with the *merR* gene removed (Figure S3). The *merOP*-*luxAB* genes or *merOP*-*sfGFP* genes were amplified by PCR and integrated into plasmid pET28a by restriction endonuclease sites *Xba*I and *Eco*RI. The *luxAB* genes, *merOP* region, and *sfGFP* gene were integrated into plasmid pET28a by *Xba*I and *Eco*RI to form vector pLPmerS (Figure S3). The promoter *P_cpc560_* and *merR* gene were integrated into plasmid pTf16 by *Sac*I and *Xho*I to form vector pPcpc560M (Figure S3). The genes *P_cpcBA_*-*sfGFP*, *P_cpc560_*-*sfGFP*, *P_psbAII_*-*sfGFP*, *P_rnpB_*-*sfGFP*, *P_tic_*-*sfGFP*, or *P_cmv_*-*sfGFP* were integrated into plasmid pET28a by restriction endonuclease sites *Xba*I and *Eco*RI to form pPcpcBAS, pPcpc560S, pPpsbAIIS, pPrnpBS, pPticS, and pPcmvS, respectively. Next, 2 μL of ligation product was transformed into 100 μL DH5α competent cells. The cells were grown for 16 h at 37 °C in Luria Broth (LB) agar plates (20 mg/L chloramphenicol for pTf16 or 30 mg/L kanamycin for pET28a). Single clones from LB agar plates were identified by PCR using Taq Master Mix. Positive samples were sent to Sangon Biotech for sequencing.

The derivatives *P_mer-M1_* to *P_mer-M17_* were generated by site-directed mutagenesis using PrimeSTAR HS DNA Polymerase. PCR amplification procedure: (1) cycle: 98 °C, 10 s; 45 °C, 5 s; 72 °C, 8 min, 15 cycle; (2) preservation: 4 °C. The final concentrations of components: (1) PrimeSTAR buffer: 1×; (2) dNTP Mix: 0.2 mM each; (3) primer: 0.25 μM each; (4) template DNA: 20 pg/μL; (5) PrimeSTAR HS DNA polymerase: 1.25 U/50 μL. The final volume of the PCR is 50 μL. After adding 0.5 μL of QuickCut™ Dpn I to 25 μL of PCR product for 20 min at 37 °C, 3 μL was transformed into 100 μL of DH5α competent cells. Bacteria were grown in LB agar plates (30 mg/L kanamycin) at 37 °C for ~16 h. Samples were sent to Sangon Biotech for sequencing. Sequences were compared using BLAST in the NCBI (National Center for Biotechnology Information) database (https://blast.ncbi.nlm.nih.gov/Blast.cgi (accessed on 15 January 2024)).

### 2.4. Luminescence Assays

The vectors pMPmerL (WT and M1–M17) and pPmerL (WT and M1–M17) were transformed into *E. coli* DH5α, respectively (Figure 1E). Single colonies were separately transferred from LB agar plates (30 mg/L kanamycin) into liquid LB medium (30 mg/L kanamycin) and incubated at 37 °C for about 16 h. The bacteria were collected by centrifuging at 5000 rpm for 5 min and the cell pellet was washed twice with M9 minimal medium (M9 medium). Subsequently, 100 µL of the cell suspension was inoculated into 5 mL of fresh M9 medium (30 mg/L kanamycin and 0 nM, 1 nM, 2 nM, 5 nM, 10 nM, 20 nM, 50 nM, or 100 nM HgCl_2_). The bacteria were incubated (~6–12 h) at 37 °C until the mid-exponential phase (the optical density was measured at 600 nm, OD600~0.5). Then, the luminescence reaction was initiated by adding 1% (*v*/*v*, using ethanol as solvent) decanal at 1:20 (*v*/*v*). The result of 50 nM mercury ions was taken as the fully induced expression level (Figure S4).

The vectors pMPmerL (WT and M1–M17) and pPcpc560M were cotransformed into *E. coli* DH5α (30 mg/L kanamycin and mg/L chloramphenicol). The subsequent steps were the same as described above except for the addition of the antibiotic chloramphenicol.

Luminescence measurements were performed in white 96-well plates with Varioskan^®^ Flash (Thermo Fisher Scientific Inc., Waltham, MA, USA). The wells were measured for 1 s, and the dynamic range was selected as “Auto Range”. The mean and standard deviation (SD) of the three colonies tested were shown and compared by Tukey HSD (Honestly Significant Differences) test. After increasing the concentration of bacteria by centrifugation (OD600 ~ 1.0), luminescence images were captured for 1.1 s using an Apple iPhone 12 Pro.

### 2.5. Fluorescence Assays

Superfolder GFP expressed by the *sfGFP* gene is stable and visible, optimized for analyzing higher expression levels. The vector pMPmerS and pPmerS (WT and M1–M17) containing *P_mer_* or their derivatives were transformed into *E. coli* DH5α, respectively (Figure 1E). Single colonies of the WT strain and its derivatives were separately transferred from LB agar plates into liquid LB medium (30 mg/L kanamycin, mercury-free) and incubated at 37 °C until the OD600 was approximately 1.0.

Fluorescence measurements were performed using Varioskan^®^ Flash in 96-well UV-transparent plates with an excitation peak at 488 nm and an emission peak at 517 nm. The wells were measured for 100 ms and the bandwidth was selected to be 12 nm. After increasing the concentration of bacteria by centrifugation (OD600 ~ 3.0), fluorescence images were captured for 30 ms using an Apple iPhone 12 Pro.

### 2.6. Data and Statistical Analysis

Luminescence and fluorescence data normalization: (sample value–background value)/OD 600. Data were collated using Excel 2021, plotted in Origin 2023b, and analyzed by one-way ANOVA with Tukey’s test using IBM SPSS Statistics 26. * *p* < 0.05, ** *p* < 0.01, *** *p* < 0.001.

### 2.7. Sodium Dodecyl Sulfate-Polyacrylamide Gel Electrophoresis Assays (SDS-PAGE)

Single colonies of the derivatives were separately transferred into LB medium, incubated at 37 °C for about 12–16 h. Then, 8 µL of overnight culture was mixed with 2 µL of 4× Protein SDS PAGE Loading Buffer (Takara) and incubated at 95 °C for 5 min. Subsequently, gel electrophoresis of proteins was performed using 5–14% polyacrylamide gel (Acrylamide:N,N′-Methylenebisacrylamide 29:1, gel size (L × W): 83 × 73 mm, comb thickness: 1.0 mm) at a constant current of 20 mA. The gels were stained with R250 and imaged with ChemiDoc™ XRS+ (Bio-Rad, Bio-Rad Laboratories, Inc., Hercules, CA, USA).

### 2.8. Quantitative Reverse Transcriptase PCR (qRT-PCR) Analysis

Single colonies of the WT strain and its derivatives (*P_mer-M10_* and *P_mer-M13_*) were picked from LB agar plates (30 mg/L kanamycin), inoculated separately into liquid LB medium with 30 mg/L kanamycin, and incubated at 37 °C for about 16 h. Subsequently, 100 µL of cell suspension was inoculated into 10 mL of fresh LB medium and incubated at 37 °C until the mid-exponential phase (OD600 of 0.5–0.6). The bacteria were collected by centrifugation at 5000 rpm for 5 min and total RNA was extracted using the Bacterial Total RNA Isolation Kit (Sangon). Reverse transcription of RNA was performed using the iScript™ cDNA synthesis kit. Real-time qPCR analysis was performed using SsoFast™ EvaGreen^®^ Supermix (Bio-Rad) on a CFX96™ Real-Time System C1000™ Thermal Cycler (Bio-Rad). The housekeeping gene 16S rRNA was used as an internal control.

### 2.9. Analyzing the Expression Activity

Bacteria containing different *mer* promoter derivatives or other constitutive promoters to express the downstream gene *sfGFP* were collected after overnight incubation in LB medium at 37 °C and 250 rpm. Subsequently, the gel electrophoresis of proteins was performed as described previously. The gels were imaged with ChemiDoc™ XRS+ (Bio-Rad) and the band intensity was analyzed Image Lab™ (Bio-Rad).

### 2.10. Electrophoretic Mobility Shift Assays (EMSA)

The construction, expression, and purification of *Tn501* MerR can be found in our previously published work [16]. DNA (Table S4) was annealed using a T100 PCR thermal cycler (Bio-Rad). Promoter DNA (30 nM) and MerR (0–600 nM) was mixed in binding buffer (10 mM Tris-HCl pH 8.0, 50 mM KCl, 5 mM MgCl_2_ and 10% glycerol) and incubated at 25 °C for 10 min before electrophoresis (80 V, 45 min) using 8% native PAGE gel.

### 2.11. Alignment of MerR Family TFs and Regulated Promoters

All crystal structures in this study were generated by PyMOL software v2.6 [69]. The MerR family TF crystals used for structural comparison were BmrR (PDB: 7CKQ) [40], CadR (PDB: 6JGX) [39], CueR (PDB: 1Q05 [70] and 6XH7 [42]), EcmrR (PDB: 6XL6) [43], MerR (PDB: 5CRL) [16], MtaN (PDB: 1R8D) [37], and PbrR (PDB: 5GPE) [71]. The crystals used to analyze the structure and interactions of the sigma factor were PDB: 5TW1 (*Mtb* AP3 promoter from *Mycolicibacterium smegmatis* MC2 155) [72], 6M7J (6M7J (PDB No.) was the *rRNA* P3 promoter and RNA polymerase from *Mycolicibacterium tuberculosis*) [73], the 7KHB (*rrnBP1* promoter and RNA polymerase from *E. coli* K-12) [74], and 8GZH (from *Synechocystis* sp. PCC 6803.).

Regulated promoters were *P_bmr_* (*Bacillus subtilis*, regulated by BmrR, CP121266.1), *P_cad_* (*P. putida*, regulated by CadR, CP097525.1), *P_cue_* (*E. coli*, regulated by CueR, CP104618.1), *P_ecmr_* (*E. coli*, regulated by EcmrR) [43], *P_mer_* (*P. aeruginosa*, regulated by MerR, CP127126.1), *P_mta_* (*B. subtilis*, regulated by MtaN, CP127278.1), and *P_pbr_* (*Cupriavidus metallidurans*, regulated by PbrR, CP046332.1). Sequences of MerR family TFs with crystal structure similarity were aligned using SnapGene software v5.3 (www.snapgene.com (accessed on 1 November 2021)). The sequence logo from the −7 to −37 region of promoters regulated by selected MerR family TFs was generated by WebLogo V2.8.2 (https://weblogo.berkeley.edu (accessed on 15 January 2024)).

## 3. Results and Discussion

### 3.1. Construction of mer Promoter Derivatives

Derivatives M1–M17 (pPmerLM1–M17, pMPmerLM1–M17, pPmerSM1–M17, and pMPmerSM1–M17) were constructed using plasmids pPmerL, pMPmerL, pPmerS, and pMPmerS as templates (Table 1 and Figure S1). All the constructed plasmids and mutations were confirmed by DNA sequencing at Sangon Biotech. The pPmerL plasmid represents the *mer* promoter^DopMerR^ expressing the *luxAB* genes, the pMPmerL plasmid represents the native *mer* promoter expressing the *luxAB* genes, the pPmerS plasmid represents the *mer* promoter^DopMerR^ expressing the *sfGFP* gene, and the pMPmerS plasmid represents the native *mer* promoter expressing the *sfGFP* gene.

### 3.2. Effect of the Spacing between the −35 and −10 Elements on P_mer_ Activity

**Figure 2:**
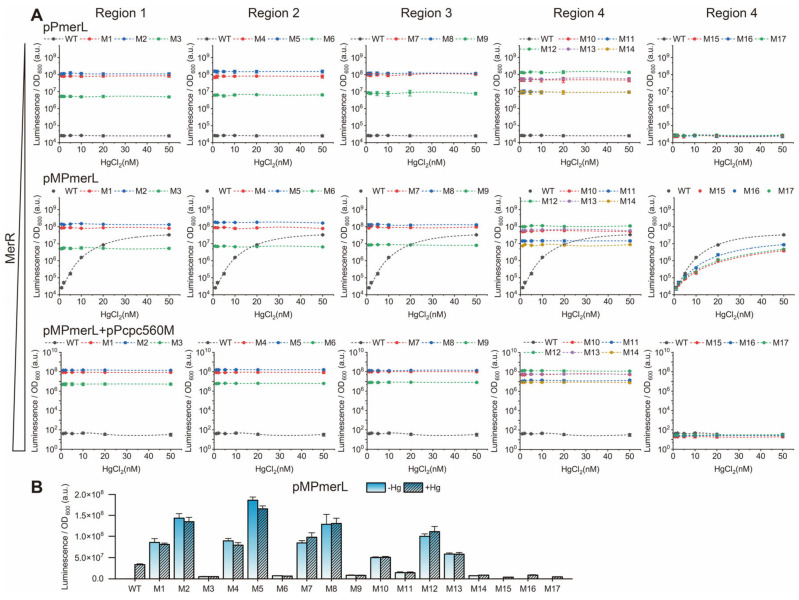
Luminescence experiments of *mer* promoters in different systems. (**A**) Luminescence of *mer* promoters (WT and derivatives M1–M17) in pPmerL, pMPmerL, and pMPmerL + pPcpc560M systems, stimulated by 0–50 nM mercury ions. Vertical coordinates are logarithmic. (**B**) Comparison of luminescence between mercury-free and 50 nM mercury ion stimulation in the pMPmerL system. Vertical scale is linear.

The regulation of *mer* promoters (WT and derivatives M1–M17) under different MerR levels was investigated using pPmerL, pMPmerL, and pMPmerL + pPcpc560M systems (Figure 2A). Luciferase expressed by the *luxAB* genes reacts with decanal to generate luminescence, which is remarkably sensitive and suitable for detecting expression levels. The results showed that MerR levels had a significant effect on the 19 bp spacer *mer* promoter (WT and derivatives M15-M17). In the absence of MerR, the promoter was not activated; at normal MerR levels, the promoter was activated by mercury ion stimulation; at ultra-high MerR levels, the promoter was not activated by the same mercury ion stimulation. Above optimal levels, the capture of mercury ions by MerR unbound to promoter DNA may interfere with the activation of transcription by promoter-bound MerR. In luminescence assays, the level of expression of the WT construct (19 bp spacer) is very low under non-induced conditions. The addition of mercury ions significantly increased expression levels only in the presence of the MerR TF. Apparently, the MerR TF and the unusual spacer length are indispensable for this specific regulation.

The shortened derivatives M1 to M14 (pPmerL, pMPmerL, and pMPmerL + pPcpc560M systems) constitutively expressed the *luxAB* genes regardless of the presence of MerR and mercury. This suggests that the Hg-MerR-regulated wild-type *mer* promoter expresses downstream genes by distorting DNA, thereby shortening the spatial distance between the −35 and −10 elements. The spatial distance between the −35 and −10 elements of the partially shortened derivative promoter (specifically M1, M2, M4, M5, M7, M8, M10, M12, and M13) is appropriate for efficient recognition by RNA polymerase, allowing downstream genes to be expressed at high levels regardless of the presence or absence of the MerR TF and mercury ion.

**Figure 3:**
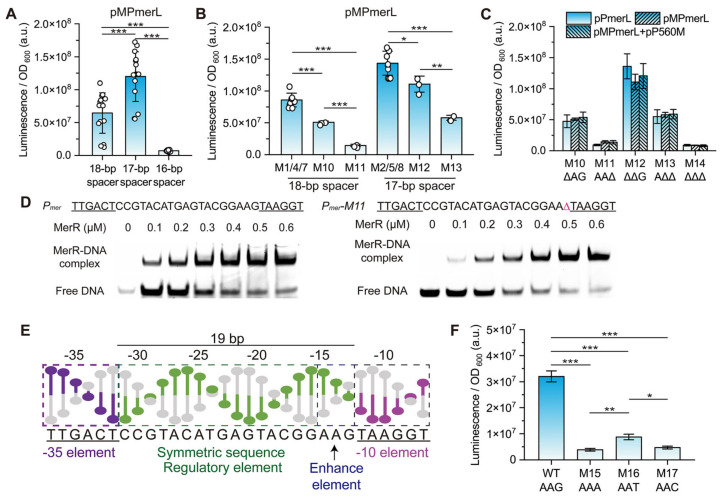
Comparison of the properties of different promoters. (**A**) Comparison of luminescence intensity from derivatives with different length spacers. Data are expressed as mean ± SD, *n* ≥ 3 independent experiments. *p*-values are determined by one-way ANOVA with Tukey test, *** *p* < 0.001. (**B**) Comparison of luminescence intensity from derivatives grouped by position or number of deleted base pairs. Data are expressed as mean ± SD, *n* ≥ 3 independent experiments. *p*-values are determined by one-way ANOVA with Tukey test, * *p* < 0.05, ** *p* < 0.01, *** *p* < 0.001. (**C**) EMSA assays for WT and M11 *mer* promoter: 30 nM for DNA and 0, 100, 200, 300, 400, 500 and 600 nM for MerR were used in the EMSAs. (**D**) Comparison of luminescence intensity of *P_mer_* derivatives M10 to M14 at different MerR levels. (**E**) Function of the region between the −35 and −10 elements. (**F**) Luminescence from derivatives substituting guanine at position −13 in the *mer* promoter. Data are expressed as mean ± SD, *n* ≥ 3 independent experiments. *p*-values are determined by one-way ANOVA with Tukey test, * *p* < 0.05, ** *p* < 0.01, *** *p* < 0.001.

Derivatives M1, M4, M7, and M10 with an 18 bp spacer (deleted −30C, −25A, −20T and −15/−14A) exhibited significantly stronger luminescence intensity than derivatives M3, M6, M9, and M14 with a 16 bp spacer (deleted ^−30^CGT^−28^, ^−25^ATG^−23^, ^−20^TAC^−18^, and ^−15^AAG^−13^), but weaker luminescence intensity than derivatives M2, M5, M8, and M12 with a 17 bp spacer (deleted ^−30^CG^−29^, ^−25^AT^−24^, ^−20^TA^−19^, and ^−15^AA^−14^) (Figure 2). The derivatives with a 16 bp spacer merely exhibited weak constitutive expression activity, suggesting that this spacing does not allow for efficient recognition by RNA polymerase. The difference in luminescence intensity could be recognized by the naked eye in the dark (Figure S5A). The average luminescence intensity from the derivatives with a 17 bp spacer (pMPmerLM2, M5, M8, M12, and M13) was 1.86-fold higher than that from the derivatives with an 18 bp spacer (pMPmerLM1, M4, M7, M10, and M11) and 16.8-fold higher than that from the derivatives with a 16 bp spacer (pMPmerLM3, M6, M9, and M14) (Figure 3A). Among these promoters, those with a 17 bp spacer were more efficiently transcribed than those with an 18 bp spacer.

Similar phenomena were observed from derivatives M1 to M14 (pPmerS, pMPmerS, and pMPmerS + pPcpc560M systems) in the fluorescence assays (Figures S5B, S6A, S7, and Table S5). The average fluorescence intensity from derivatives with a 17 bp spacer (pMPmerSM2, M5, M8, M12, and M13) was 1.89-fold higher than that from derivatives with an 18 bp spacer (pMPmerSM1, M4, M7, M10, and M11) and 11.3-fold higher than that from derivatives with a 16 bp spacer (pMPmerSM3, M6, M9, and M14) (Figure S6B). Although there were slight fluctuations between the results of the luminescence and fluorescence assays, the relative intensities and trends were consistent. In addition, SDS-PAGE assays were further performed to eliminate possible errors between sfGFP expression levels and fluorescence intensity, such as those caused by protein misfolding (Figure S7). The results were in agreement with each other and demonstrated that spacer length was an important factor affecting the activity of *P_mer_*. The optimal spacer length for the expression of the target gene by *P_mer_* was 17 bp, followed by 18 bp and 16 bp. The 17–18 bp spacer *mer* promoter derivative had strong constitutive expression activity (Figure S8). MerR activated *P_mer_* by generating an equivalent contraction of the spacing between the −10 and −35 elements to the deletion of base pairs, once again supporting the DNA distortion mechanism regulated by MerR.

However, the model of spacer length regulating the expression level of target genes could not independently explain the similarity in expression activity of the 18 bp spacer derivative M10 (deleted −15/−14A) and the 17 bp spacer derivative M13 (deleted ^−14^AG^−13^) (Figure S6C). Moreover, the similarity between *P_mer_* derivative M10 and derivative M11 is not only in expression activity but also in transcriptional activity (Figure S9 and Table S6). This suggests that the abnormally long spacer region of *P_mer_* might have an additional function in the regulatory process besides the conformational distortion regulated by MerR.

### 3.3. Role of Different Positions in the Spacer between the −35 and −10 Elements of P_mer_

Next, the expression activity of derivatives with the same spacer length but with base pair deletions at different positions in the spacer region was compared to explore the relationship between sequences at different positions and *mer* promoter activity. As derivatives with a spacer length of 18 bp, the expression level (luminescence/fluorescence) from derivative M7 (304%/302%) > M1 (253%/270%) ~ M4 (247%/264%) > M10 (158%/178%) > M11 (45%/46%); as derivatives with a spacer length of 17 bp, the expression level from derivative M5 (517%/540%) > M2 (421%/454%) ~ M8 (407%/435%) > M12 (346%/374%) > M13 (181%/198%); and as derivatives with a spacer length of 16 bp, the expression levels from them was too low to be compared confidently (Table 1 and Figure 2).

**Figure 4:**
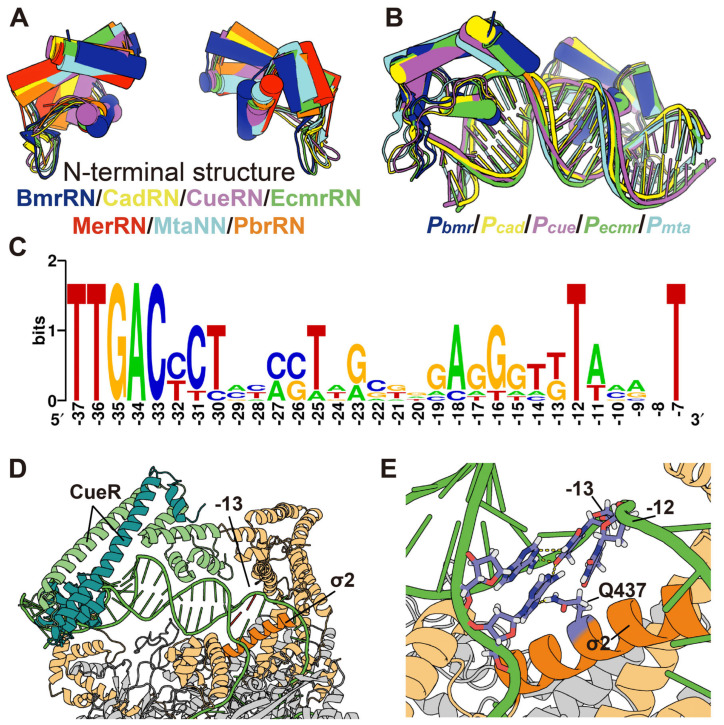
Alignment of the promoter −13 base. (**A**) Similarity in the N-terminal structure of MerR family transcription factors. BmrR (PDB: 7CKQ) is blue, CadR (PDB: 6JGX) is yellow, CueR (PDB: 1Q05) is purple, EcmrR (PDB: 6XL6) is green, MerR (PDB: 5CRL) is red, MtaN (PDB: 1R8D) is cyan, and PbrR (PDB: 5GPE) is orange. (**B**) Structure of promoter DNA distortion regulated by MerR family transcription factors. (**C**) Sequence alignment between the −10 and −35 elements of promoters regulated by MerR family TFs. (**D**) Complex crystals of activated forms of CueR (a homologue of MerR), promoter DNA, and RNA polymerase holoenzyme (PDB: 6XH7). CueR is indicated by the line, the sigma factor is yellow, the promoter DNA is green, and the σ2 is orange. The RNA polymerase is grey. (**E**) Interactions of base pair at position −13 in the promoter regulated by CueR. The sigma factor is yellow, the promoter DNA is green, the σ2 is orange, and the RNA polymerase is grey. The base pairs at positions −12 and −13 in the promoter and the glutamine at position 437 (Q437) in σ2 are marked in purple. The yellow dotted lines show their interactions.

For derivatives with base pair deletions in the MerR-binding symmetrical sequence region (^−31^CCGTACATGAGTACGG^−16^), there was no significant difference in expression activity between them if the spacer length was equal. At the same spacer length, the expression activity of derivatives with base pairs deleted in the symmetrical sequence was several times higher than that of derivatives with base pairs deleted in the region upstream of −10 (Figure 3B and Figure S6D). Since no crystals of the *Tn501* MerR–DNA complex have been reported and the N-terminal sequences of the MerR family TFs bound to promoter DNA are similar (Figure 4A and Figure S10), crystals of other MerR family TFs in complex with promoter DNA (19 bp spacer) were analyzed as substitutes to characterize the structure of the spacer region. The promoter DNA was distorted around the center of the symmetrical sequence, forming a region with significant conformational differences from the B-form (Figure 4B and Figure S11). On the other hand, the region upstream of −10 (−15 to −13) maintains a localized conformation similar to B-form DNA, although it has changed position due to the distortion [45]. This means that sequence length plays a major role in regulating expression activity in the symmetrical sequence region, exactly where the promoter DNA is distorted, and in the region upstream of −10, there are additional determinants beyond sequence length involved in the regulation of expression activity.

Significantly, the expression activity of derivative M10 (deletion of −15/−14A) was 3.51-fold/3.86-fold (luminescence/fluorescence assay) higher than that of M11 (deleted −13G) at the same spacer lengths; and the expression activity of derivative M12 (deleted ^−15^AA^−14^) was 1.91-fold/1.89-fold higher than that of M13 (deleted ^−14^AG^−13^) (Figure 3A,B). This highlighted the importance of −13G in the region upstream of −10 for the high-level expression of the target gene compared to the other positions. This finding explained the enigma of the similarity in expression activity between derivatives M13 (deleted ^−14^AG^−13^) and M10 (deleted −15/−14A): the 17 bp spacer in derivative M13 favored higher expression activity compared to the 18 bp spacer in derivative M10, whereas the deletion of −13G in derivative M13, in turn, reduced expression activity.

### 3.4. Role and Effects of the Regulatory Factor MerR and merR Gene

Luminescence of the derivative was detected in the pLPmerS system to study the effect of mutations in the spacer region on *merR* gene expression levels. The results showed that the expression level of the *merR* gene in the derivatives basically maintained that of the wild type (Figure S12), suggesting that the differences in expression activity of the derivatives were not caused by *merR* gene expression levels.

The EMSA assay was performed to determine the affinity of MerR for DNA with different *mer* promoters (WT and mutants M1, M4, M7, M10, M11, M12, and M14). The results showed that deletion (M1, M10, M11, M12, and M14) of the sites not within the “^−29^GTACXXXXGTAC^−18^” sequence had little effect on the binding of MerR to promoter DNA (Figure 3C and Figure S13). The deletion (M4 and M7) of sites within the “^−29^GTACXXXXGTAC^−18^” sequence prevented MerR from binding to promoter DNA. Derivatives with similar expression activity had different affinities of their promoter DNA for MerR. It suggested that the difference in activity of the derivatives was not caused by the affinity of promoter DNA for MerR. MerR levels had no significant effect on the 16–18 bp spacer *mer* promoters (derivatives M1–M14), although it still bound to some promoters (Figure 2A, Figures S7 and S13) whereas changes in activity caused by the −13G site were present in all systems (Figure 3D and Figure S6E), suggesting that −13G upstream of −10 acted as a determinant by interacting with the RNA polymerase holoenzyme and not MerR.

### 3.5. Conservation and Function of Guanine at Position −13 (−13G) in P_mer_

Our findings suggested that the abnormally long 19 bp spacer (−31 to −13) in *P_mer_* had two functions, one for MerR recognition and binding (−31 to −16) and the other for increasing downstream gene expression levels (−15 to −13) (Figure 3E). The role of guanine at position −13 (−13G) in the −15 to −13 region was critical. To explore the specificity of −13G, the derivatives pMPmerLM15 (G−13A), M16 (G−13T), and M17 (G−13C) were constructed. The fully induced expression level from the wild type (−13G) was 8.3-fold, 3.7-fold, and 6.7-fold higher than that from derivatives M15, M16, and M17 (Figure 3F). This result established that guanine at position −13 of *P_mer_* was specific to high expression levels of target genes.

The alignment of the promoter sequence regulated by the MerR family TFs with the 19 bp spacer between the −10 and −35 elements revealed that the bases at the −13 position were predominantly thymine and less often guanine (Figure 4C and Figure S14). However, −13G was not conserved in all promoters, since these promoters are recognized by different MerR family transcription factors responding to different environmental stimuli that are not equally toxic to cells. Correspondingly, the expression level from the derivative M16 (G−13T) was 2.3-fold higher than that from M15 (G−13A) and 1.8-fold higher than that from M17 (G−13C). This indicated that the −13 base (T or G) in the promoter controlled by MerR family TFs contributed to the high expression level of the target genes. MerR family TFs regulate promoters with unusually long spacers. Notably, the symmetric sequence to which the TF binds is not centered between the −35 and −10 elements but is biased toward the −35 element.

To provide the possibility that the base at position −13 interacts with the RNA polymerase holoenzyme, we performed a crystal structure analysis. The most conserved region 2 (σ_2_) of sigma factor 70 (σ^70^) was divided into four subregions; subregion 2.3 (σ_2.3_) is implicated in DNA melting, and subregion 2.4 (σ_2.4_) is associated with recognizing the −10 element [54,75]. An α-helix containing a sequence ^430^YATWWIRQAITRSIAD^445^ in σ_2.3_ to σ_2.4_ interacts with the −10 element of the promoter. Q437, which is known to bind the base at position −12, formed hydrogen bonds between the base pairs at positions −12 and −13 in partial crystals (Figure S15). Since MerR proteins lack corresponding complex crystals, we substituted CueR for MerR. Similar interactions were present in a complex crystal of the MerR homologue CueR (Figure 4D,E). Hydrogen bonding occurred between the base pairs at positions −12 (−12A’) and −13 (−13T) in the promoter and the glutamine at position 437 (Q437). Thus, it was hypothesized that −13G enhances the expression activity of *P_mer_* precisely by interacting with the base pair −12T/A and the Q437 residue in σ_2.4_. In addition, both guanine and thymine contain secondary amide to form hydrogen bonds for base complementary pairing, the amide of guanine being more protruding to facilitate the formation of “mismatches” with the adenine in −12T/A (Figure S16).

## 4. Conclusions

In this paper, we analyzed the effect of the mutant spacer sequence between the −35 and −10 elements on the expression activity of the *mer* promoter by double-calibrated semi-quantitative analysis of luminescence and fluorescence assays. The results showed that (1) derivatives with a 3 bp deletion (16 bp spacer) had weak constitutive activity; (2) derivatives with a 1–2 bp deletion (18–17 bp spacer) were generally strong constitutive promoters; (3) 17 bp derivatives were generally stronger than 18 bp ones as well as the MerR-activated wild-type; (4) the 19 bp promoter was regulated by MerR and the constitutive expression activity of the 16–18 bp promoter was almost unaffected by MerR; (5) the derivatives retaining the sequence “^−15^AAG^−13^” in the upstream region of −10 showed higher expression activity at the same spacer length; (6) the activated wild-type promoter (−13G) was higher than that of the activated derivatives (G−13A, G−13T and G−13C).

The spacer deletion mutants M10 (deleted −15/−14A) and M13 (deleted ^−14^AG^−13^) in previous studies had similar expression activity, as the deletion of −13G partially counteracted the increase in activity caused by spacer shortening. The symmetrical sequence in the spacer region of the *mer* promoter, as well as other promoters regulated by MerR family TFs, was not centered between the −35 and −10 elements but was biased toward the −35 element. The base sequence upstream of the −10 element in the ultra-long spacer was not used for transcription factor binding but had additional functions. Compared to the common TG (^−15^TG^−14^) extension promoters, −13G had a different position and sequence, but both interacted with the sigma factor. Crystal structure analysis provided spatial opportunities for interactions.

Our findings support that spacer length and −13G in the region upstream of −10 work synergistically in regulating downstream gene expression levels. This study advances the understanding of the process by which the *mer* promoter modulates the expression levels of *mer* genes.

## Figures and Tables

**Table 1:** Relative expression levels from *P_mer_* and *P_mer_* derivatives.

Promoter	Expression Level (%) ^1^			
	pPmerL (−MerR)		PMPmerL (+MerR)	
	Without Hg (II)	With Hg (II)	Without Hg (II)	With Hg (II)
*P_mer-WT_*	0	0	0	100
*P_mer-M1_*	264	266	267	253
*P_mer-M2_*	345	344	447	421
*P_mer-M3_*	17	15	16	17
*P_mer-M4_*	222	246	279	247
*P_mer-M5_*	500	484	582	517
*P_mer-M6_*	19	20	22	20
*P_mer-M7_*	306	332	264	304
*P_mer-M8_*	376	373	401	407
*P_mer-M9_*	27	24	26	25
*P_mer-M10_*	142	142	156	158
*P_mer-M11_*	30	10	45	45
*P_mer-M12_*	126	128	311	346
*P_mer-M13_*	75	73	182	181
*P_mer-M14_*	28	28	23	27
*P_mer-M15_*	0	0	0	12
*P_mer-M16_*	0	0	0	27
*P_mer-M17_*	0	0	0	15

^1^ The data were averaged from at least three individual experiments. Expression levels were normalized to the percentage of fully induced (50 nM HgCl_2_) expression levels of the wild type (*P_mer_*).

## Data Availability

Data are contained within the article.

## Supplementary Materials

The following supporting information can be downloaded at: https://www.mdpi.com/article/10.3390/genes15040490/s1, Figure S1 Sequence and spacer length of *mer* promoter derivatives. The −35 element is purple and the −10 element is pink. Mutation sites are marked with dotted boxes on a yellow background. Figure S2 Scheme of the DNA conformation of the region between the −35 and −10 elements of *mer* promoter derivatives. The −35 element is purple, the −10 element is pink, and the intervening spacer is green. Figure S3 Plasmid maps of the vectors constructed in this study. (A) pPmerL, (B) pMPmerL, (C) pPmerS, (D) pMPmerS, (E) pLPmerS, (F) pPcpc560M. Figure S4 Mercury ion concentration affects bacterial growth and luminescence intensity. (A) Toxicity of mercury ions (0–100 μM) to *Escherichia coli*. (B) Toxicity of mercury ions (0-100 nM) to *E. coli* containing the pMPmerL plasmid at different times. (C) Mercury ions (0-100 nM) induced luminescence in *E. coli* containing the pMPmerL plasmid (linear coordinates). (D) Mercury ions (0-100 nM) induced luminescence in *E. coli* containing the pMPmerL plasmid (logarithmic coordinates). Figure S5 Visualization of derivative expression activity. (A) Visualization of the difference in luminescence levels between the derivatives M1-M14 of the pMPmerL plasmid. (B) Visualization of the difference in fluorescence levels between the derivatives M1-M14 of the pMPmerS plasmid. Figure S6 Expression activity of *mer* promoter derivatives. (A) Fluorescent gene expression levels from derivatives M1 to M14 of the pMPmerL plasmid. (B) Comparison of fluorescence intensity from derivatives with different length spacers. (C) Similarity of promoter activity between derivative M10 and derivative M13. (D) Comparison of fluorescence intensity of derivatives grouped by position or number of deleted base pairs. (E) Comparison of fluorescence intensity of *P_mer_* derivatives M10 to M14 in the presence and absence of MerR. Figure S7 The sfGFP expression levels from derivatives M1-M17 without mercury in three different systems (pPmerS, pMPmerS and pMPmerS+pPcpc560M). Figure S8 Expression levels of sfGFP in *E. coli* from different promoters analyzed with Image Lab™ (Bio-Rad). Lanes 1 to 8 are promoters *P_cpcBA_* (*cpcBA* promoter of *Synechocystis* sp. 6803, strong) [76], *P_cpc560_* (*cpc560* promoter of *Synechocystis* sp. 6803, strong) [63], *P_mer_*-M10 (*merT* promoter mutant M10 of *Pseudomonas aeruginosa,* strong), *P_mer_*-M13 (*merT* promoter mutant M13 of *Pseudomonas aeruginosa,* strong), *P_psbAII_* (*psbAII* promoter of *Synechocystis* sp. 6803) [64], *P_rnpB_* (*rnpB* promoter of *Synechocystis* sp. 6803) [65], *P_tic_* (*tic* promoter of *Synechocystis* sp. 6803) [66] and *P_cmv_* (*cmv* promoter of Human betaherpesvirus) [67]. Figure S9 Transcript levels of gene (*luxA* or *sfGFP*) downstream of the *mer* promoter (WT, derivatives M10 or M13) (A) RT-PCR assays for *luxA* gene. (B) RT-PCR assays for *sfGFP* gene. The control group was S16 rRNA. Transcript levels of the wild-type *mer* promoter are shown in orange, mutant M10 in cyan, and mutant M10 in slateblue. Figure S10 Alignment of N-terminal sequences of MerR family transcription factors. BmrR (*Bacillus subtilis*, WP_003230325.1), CadR (*Pseudomonas putida*, WP_198743526.1), CueR (*Escherichia coli*, WP_089632711.1), EcmrR (*Escherichia coli*, WP_053276550.1), MerR (*Pseudomonas aeruginosa*, WP_003131969.1), MtaN (*Bacillus subtilis*, WP_038829835.1), PbrR (*Cupriavidus metallidurans*, WP_134593310.1). Figure S11 Schematic comparison of the B-form promoter DNA and distorted promoter DNA. Non-activated B-form is red, activated distortion-form is green. Figure S12 Expression levels of the merR gene from wild type and different derivatives. Figure S13 MerR binding to promoter DNA of wild type and derivatives. Non-activated B-form is red, activated distortion-form is green. The promoter DNA fragments contain −35 to −10 elements and their spacer length ranges from 16-19 bp. 30 nM for DNA and 0, 100, 200, 300, 400, 500 and 600 nM for MerR were used in the final EMSA experiment. Figure S14 Sequence alignment of promoters with 19 bp spacer between −10 and −35 elements regulated by MerR family TFs. The −35 and −10 elements are marked with a double underline. Symmetrical sequences are shown in bold. BmrR (*Bacillus subtilis*, *P_bmr_*, CP121266.1), CadR (*Pseudomonas putida*, *P_cad_*, CP097525.1), CueR (*Escherichia coli*, *P_cue_*, CP104618.1), EcmrR (*Escherichia coli*, *P_ecmr_*) [43], MerR (*Pseudomonas aeruginosa*, *P_mer_*, CP127126.1), MtaN (*Bacillus subtilis*, *P_mta_*, CP127278.1), PbrR (*Cupriavidus metallidurans*, *P_pbr_*, CP046332.1). Figure S15 Contact of region 2 (σ2) in sigma factor 70 (σ70) with promoter positions −13 and −12 in various RNAP holoenzyme complex crystals. The promoter DNA is green, the sigma factor is yellow and the σ2 is orange. The base pairs at positions −12 and −13 in the promoter and the glutamine at position 437 (Q437) in σ2 are marked in purple. The yellow dotted lines show their interactions (hydrogen bonding). 5TW1 (PDB No.) is *Mtb* AP3 promoter from *Mycolicibacterium smegmatis* MC2 155 [72]. 6M7J (PDB No.) is *rrnA* P3 promoter and RNA polymerase from *Mycobacterium tuberculosis* [73]. 7KHB (PDB No.) is *rrnBP1* promoter and RNA polymerase from *Escherichia coli* K-12 [74]. 8GZH (PDB No.) is from *Synechocystis* sp. PCC 6803. Figure S16 Schematic structures of the four bases and common functional groups of guanine and thymine. Secondary amides, which form hydrogen bonds in guanine and thymine, are labelled in red. Table S1 Promoter sequences. Table S2 Primers for the template plasmid construction. Table S3 Mutagenic primers for the *mer* promoter. Table S4 DNA for the electrophoretic mobility shift assay (EMSA). Table S5 Expression activity of *mer* promoter derivatives in the pMPmerS plasmid. Table S6 Primers for Reverse-transcription PCR (RT-PCR) assay.
