# Supplementary material for: Determinants of mer Promoter Activity from Pseudomonas aeruginosa
Source: Genes (Basel). 2024 Apr 13;15(4):490. doi: 10.3390/genes15040490 (PMC11049809; doi:10.3390/genes15040490)
Supplement: Supplementary file 1 [file genes-15-00490-s001.zip › genes-2946534-supplementary.pdf]

Supplementary Materials:

Determinants of *mer* promoter activity from *Pseudomonas aeruginosa*

*Qingyuan Hu, Jue Wang, Chunhong Liu, Yu Feng, Hao Chen**

State Key Laboratory of Coordination Chemistry, School of Chemistry and Chemical Engineering, Nanjing University, 210023 Nanjing, China; huqingyuan1991@163.com (Q.H.); wj690189386@163.com (J.W.);
liuchunhong1111@126.com (C.L.); 602023240027@smail.nju.edu.cn (Y.F.)
* Correspondence: E-mail: chenhao@nju.edu.cn.

Keywords: *mer* promoter, expression activity, spacer length, position −13, guanine, synergistic regulation

Contents

Supporting Figures

Fig.S1 Sequence and spacer length of *mer* promoter derivatives.

Fig.S2 Schematic representation of DNA conformation in the region between the −35 and −10 elements for *mer* promoter derivatives.

Fig.S3 Plasmid maps of the vectors constructed in this study.

Fig.S4 Mercury ion concentration affects bacterial growth and luminescence intensity.

Fig.S5 Visualization of derivative expression activity.

Fig.S6 Expression activity of *mer* promoter derivatives.

Fig.S7 The sfGFP expression levels from derivatives M1-M17 without mercury in three different systems (pPmerS, pMPmerS and pMPmerS+pPcpc560M).

Fig.S8 Expression levels of sfGFP in *E. coli* from different promoters analyzed with Image Lab™ (Bio-Rad).

Fig.S9 Transcript levels of gene (*luxA* or *sfGFP*) downstream of the *mer* promoter (WT, derivatives M10 or M13).

Fig.S10 Alignment of N-terminal sequences of MerR family transcription factors.

Fig.S11 Schematic comparison of the B-form promoter DNA and distorted promoter DNA.

Fig.S12 Expression levels of the merR gene from wild type and different derivatives.

Fig.S13 MerR binding to promoter DNA of wild type and derivatives.

Fig.S14 Sequence alignment of promoters with 19 bp spacer between −10 and −35 elements regulated by MerR family TFs.

Fig.S15 Contact of region 2 (σ2) in sigma factor 70 (σ70) with promoter positions −13 and −12 in various RNAP holoenzyme complex crystals.

Fig.S16 Schematic structures of the four bases and common functional groups of guanine and thymine.

Supporting Tables

Table S1 Primers for the template plasmid construction.

Table S2 Mutagenic primers for the *mer* promoter.

Table S3 DNA for the electrophoretic mobility shift assay (EMSA).

Table S4 Expression activity of *mer* promoter derivatives in the pMPmerS plasmid.

Table S5 Primers for Reverse-transcription PCR (RT-PCR) assay.

Table S6 Promoter sequences.

Reference

Supporting Figures

	Mutation position		Spacer length
**Previous studies**			
	−31 or −30	ATCGCTTGACTCΔGTACATGAGTACGGAAGTAAGGTTACGCTATCCAA	18 bp
	−24	ATCGCTTGACTCCGTACAΔGAGTACGGAAGTAAGGTTACGCTATCCAA	18 bp
	−18	ATCGCTTGACTCCGTACATGAGTAΔGGAAGTAAGGTTACGCTATCCAA	18 bp
	−15 or −14	ATCGCTTGACTCCGTACATGAGTACGGAΔGTAAGGTTACGCTATCCAA	18 bp
	−14 to −13	ATCGCTTGACTCCGTACATGAGTACGGAΔΔTAAGGTTACGCTATCCAA	17 bp
**This study**			
P_{mer}	Wild type	ATCGCTTGACTCCGTACATGAGTACGGAAGTAAGGTTACGCTATCCAA	19 bp
$P_{mer\text{-}M1}$	−31 or −30	ATCGCTTGACTCΔGTACATGAGTACGGAAGTAAGGTTACGCTATCCAA	18 bp
$P_{mer\text{-}M2}$	−30 to −29	ATCGCTTGACTCΔΔTACATGAGTACGGAAGTAAGGTTACGCTATCCAA	17 bp
$P_{mer\text{-}M3}$	−30 to −28	ATCGCTTGACTCΔΔΔACATGAGTACGGAAGTAAGGTTACGCTATCCAA	16 bp
$P_{mer\text{-}M4}$	−25	ATCGCTTGACTCCGTACΔTGAGTACGGAAGTAAGGTTACGCTATCCAA	18 bp
$P_{mer\text{-}M5}$	−24 to −25	ATCGCTTGACTCCGTACΔΔGAGTACGGAAGTAAGGTTACGCTATCCAA	17 bp
$P_{mer\text{-}M6}$	−23 to −25	ATCGCTTGACTCCGTACΔΔΔAGTACGGAAGTAAGGTTACGCTATCCAA	16 bp
$P_{mer\text{-}M7}$	−20	ATCGCTTGACTCCGTACATGAGΔACGGAAGTAAGGTTACGCTATCCAA	18 bp
$P_{mer\text{-}M8}$	−19 to −20	ATCGCTTGACTCCGTACATGAGΔΔCGGAAGTAAGGTTACGCTATCCAA	17 bp
$P_{mer\text{-}M9}$	−18 to −20	ATCGCTTGACTCCGTACATGAGΔΔΔGGAAGTAAGGTTACGCTATCCAA	16 bp
$P_{mer\text{-}M10}$	−15 or −14	ATCGCTTGACTCCGTACATGAGTACGGΔAGTAAGGTTACGCTATCCAA	18 bp
$P_{mer\text{-}M11}$	−13	ATCGCTTGACTCCGTACATGAGTACGGAAΔTAAGGTTACGCTATCCAA	18 bp
$P_{mer\text{-}M12}$	−15 to −14	ATCGCTTGACTCCGTACATGAGTACGGΔΔGTAAGGTTACGCTATCCAA	17 bp
$P_{mer\text{-}M13}$	−14 to −13	ATCGCTTGACTCCGTACATGAGTACGGAΔΔTAAGGTTACGCTATCCAA	17 bp
$P_{mer\text{-}M14}$	−15 to −13	ATCGCTTGACTCCGTACATGAGTACGGΔΔΔTAAGGTTACGCTATCCAA	16 bp
$P_{mer\text{-}M15}$	−13	ATCGCTTGACTCCGTACATGAGTACGGAAATAAGGTTACGCTATCCAA	19 bp
$P_{mer\text{-}M16}$	−13	ATCGCTTGACTCCGTACATGAGTACGGAATTAAGGTTACGCTATCCAA	19 bp
$P_{mer\text{-}M17}$	−13	ATCGCTTGACTCCGTACATGAGTACGGAACTAAGGTTACGCTATCCAA	19 bp
		5'- −35 element −10 element -3'	

Fig.S1 Sequence and spacer length of *mer* promoter derivatives. The −35 element is purple and the −10 element is pink. Mutation sites are marked with dotted boxes on a yellow background.

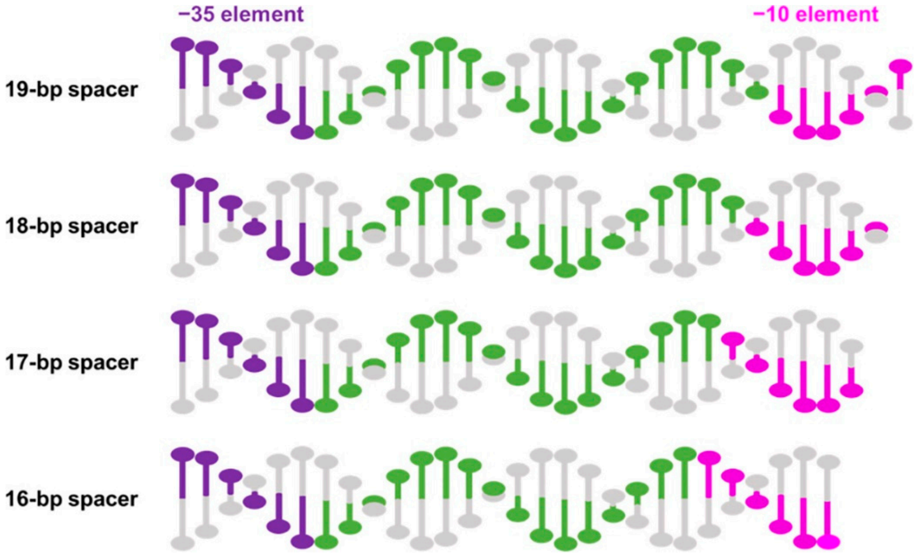

Fig.S2 Scheme of the DNA conformation of the region between the −35 and −10 elements of *mer* promoter derivatives. The −35 element is purple, the −10 element is pink, and the intervening

spacer is green.

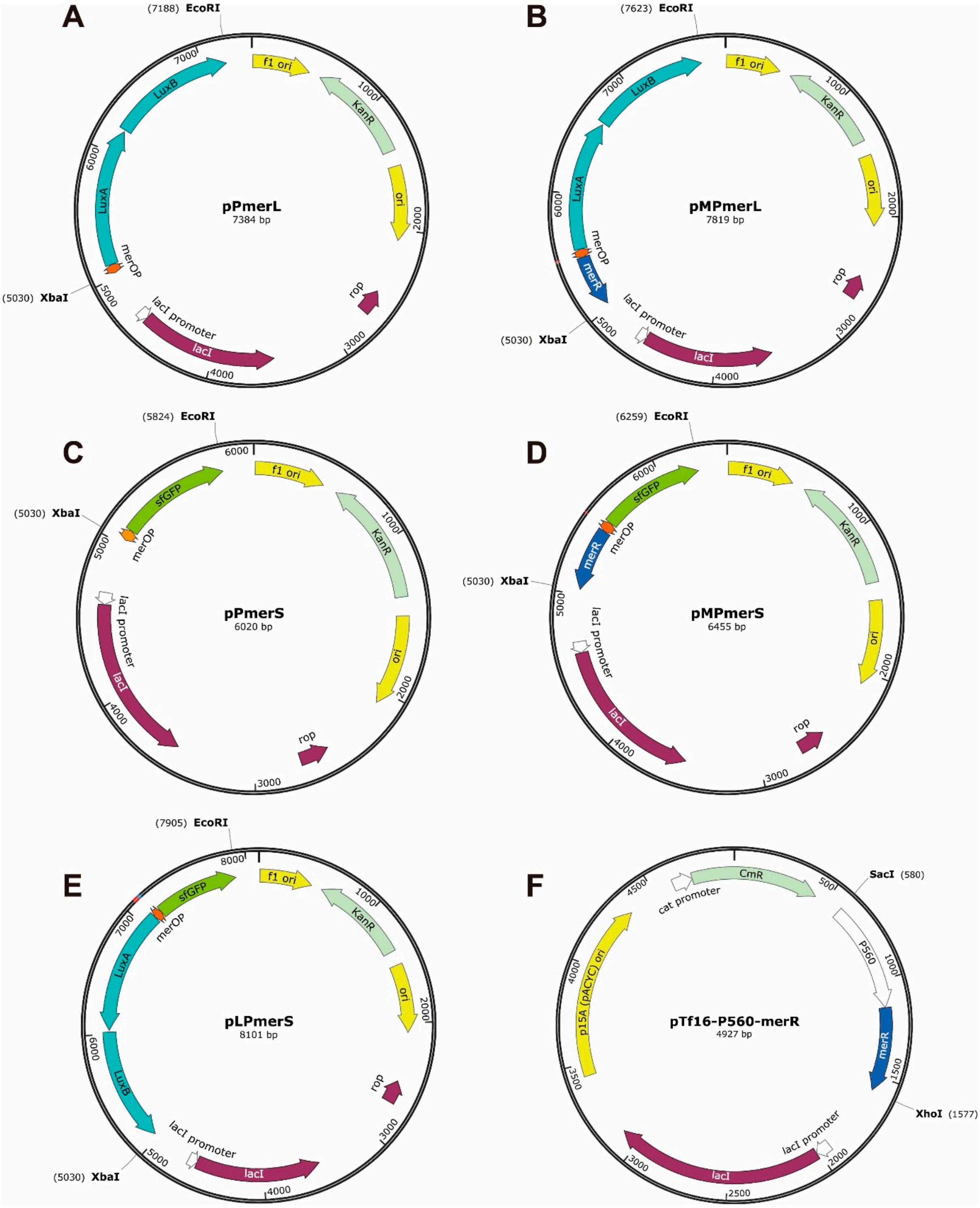

Fig.S3 Plasmid maps of the vectors constructed in this study. (A) pPmerL, (B) pMPmerL, (C) pPmerS, (D) pMPmerS, (E) pLPmerS, (F) pPcpc560M.

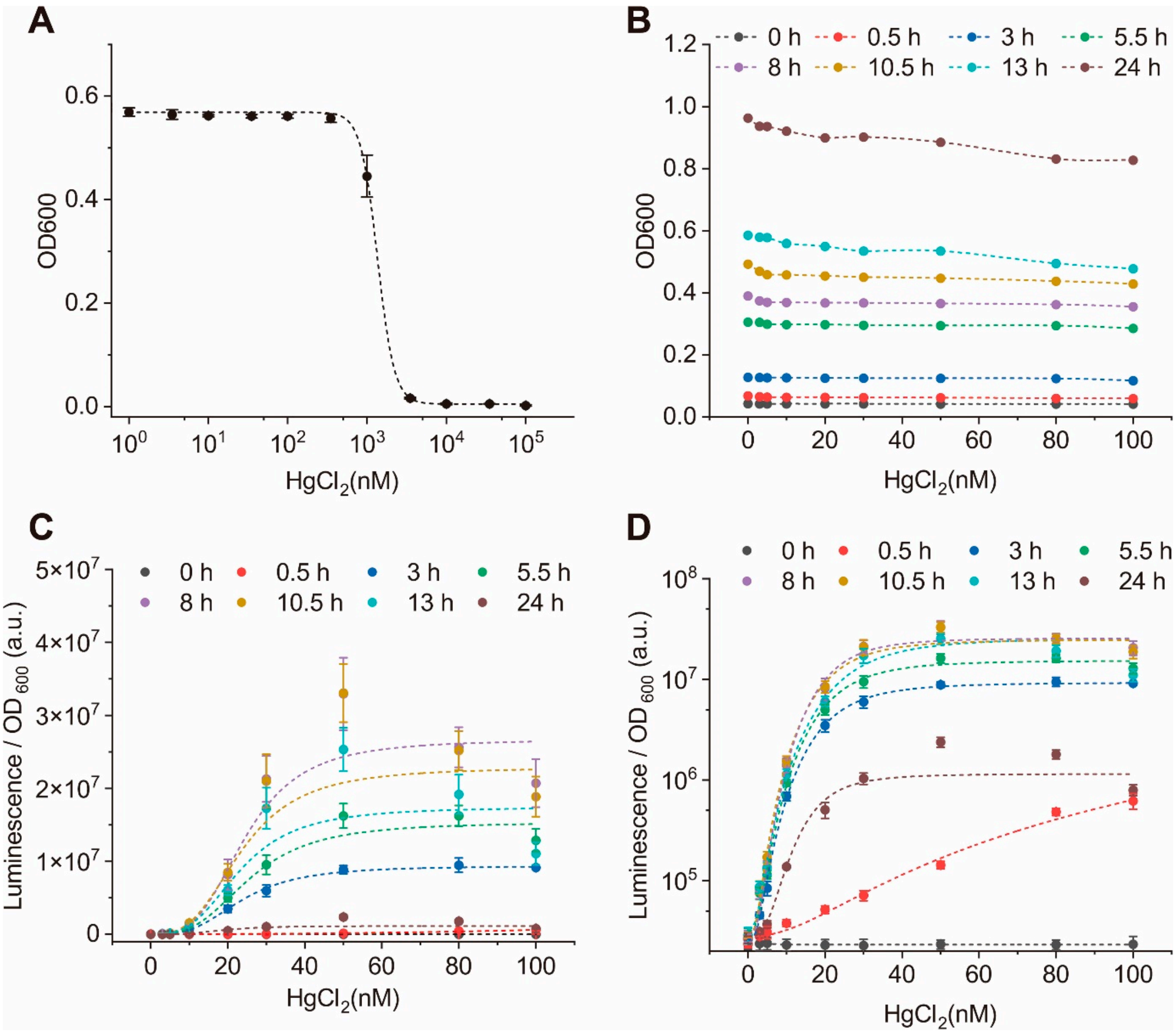

Fig.S4 Mercury ion concentration affects bacterial growth and luminescence intensity. (A) Toxicity of mercury ions (0-100 μM) to E*scherichia coli*. (B) Toxicity of mercury ions (0-100 nM) to *E. coli* containing the pMPmerL plasmid at different times. (C) Mercury ions (0-100 nM) induced luminescence in *E. coli* containing the pMPmerL plasmid (linear coordinates). (D) Mercury ions (0-100 nM) induced luminescence in *E. coli* containing the pMPmerL plasmid (logarithmic coordinates).

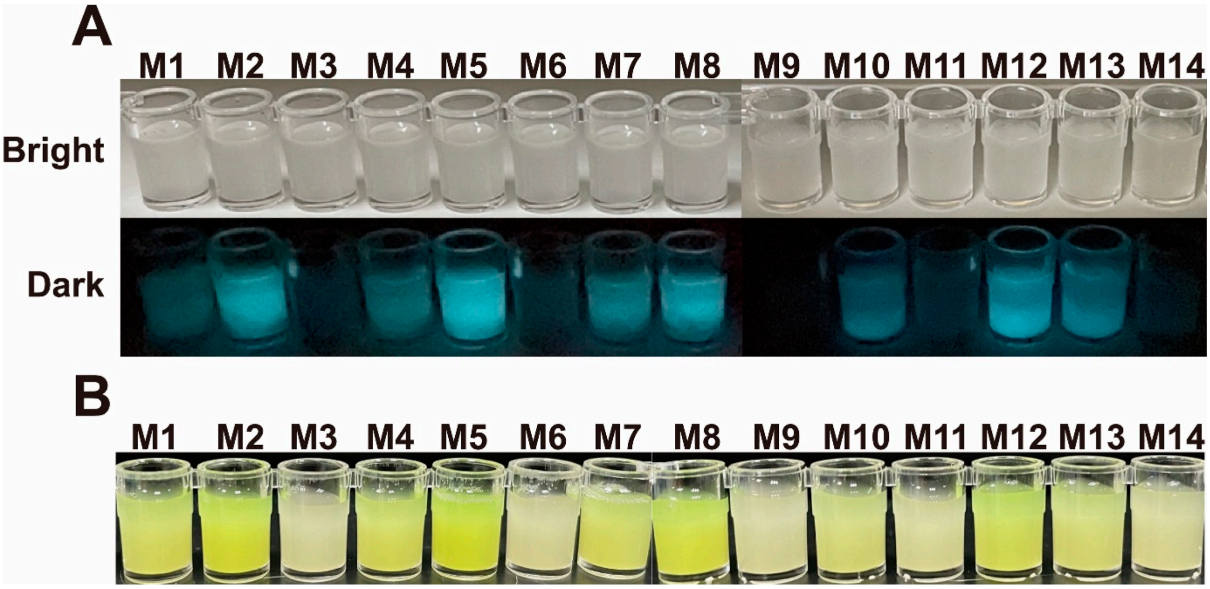

Fig.S5 Visualization of derivative expression activity. (A) Visualization of the difference in luminescence levels between the derivatives M1-M14 of the pMPmerL plasmid. (B) Visualization of the difference in fluorescence levels between the derivatives M1-M14 of the pMPmerS plasmid.

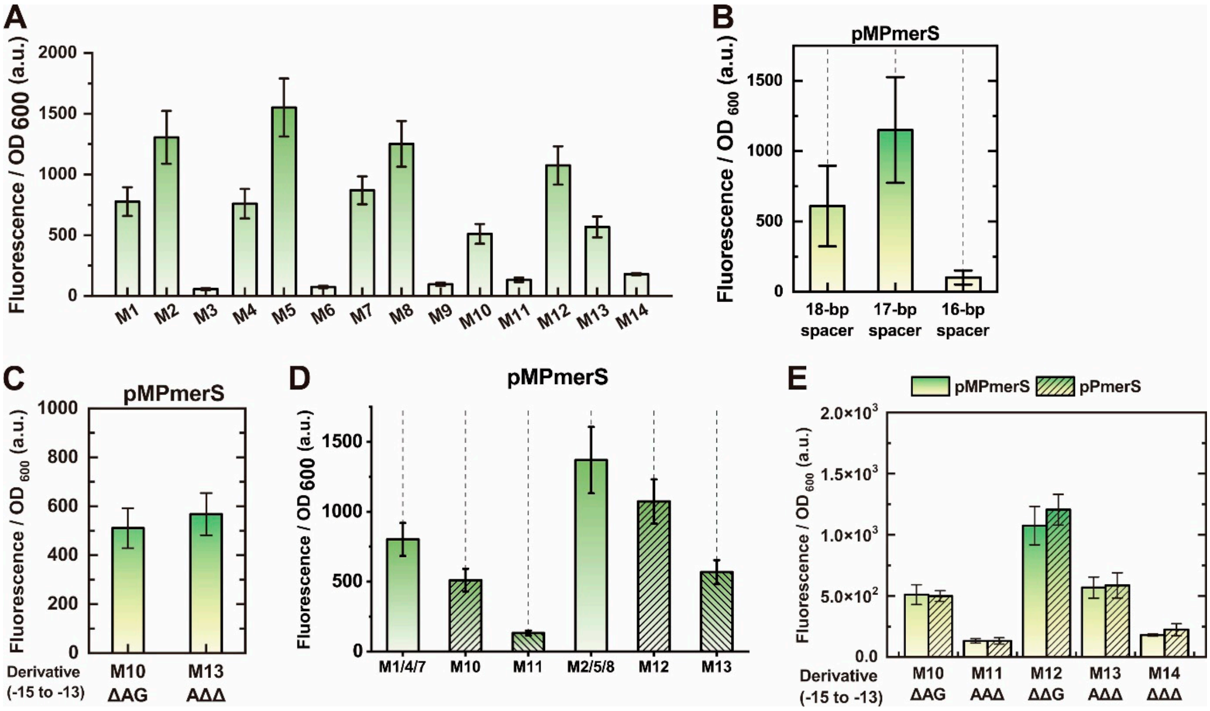

Fig.S6 Expression activity of *mer* promoter derivatives. (A) Fluorescent gene expression levels from derivatives M1 to M14 of the pMPmerL plasmid. (B) Comparison of fluorescence intensity from derivatives with different length spacers. (C) Similarity of promoter activity between derivative M10 and derivative M13. (D) Comparison of fluorescence intensity of derivatives grouped by position or number of deleted base pairs. (E) Comparison of fluorescence intensity of P_{mer} derivatives M10 to M14 in the presence and absence of MerR.

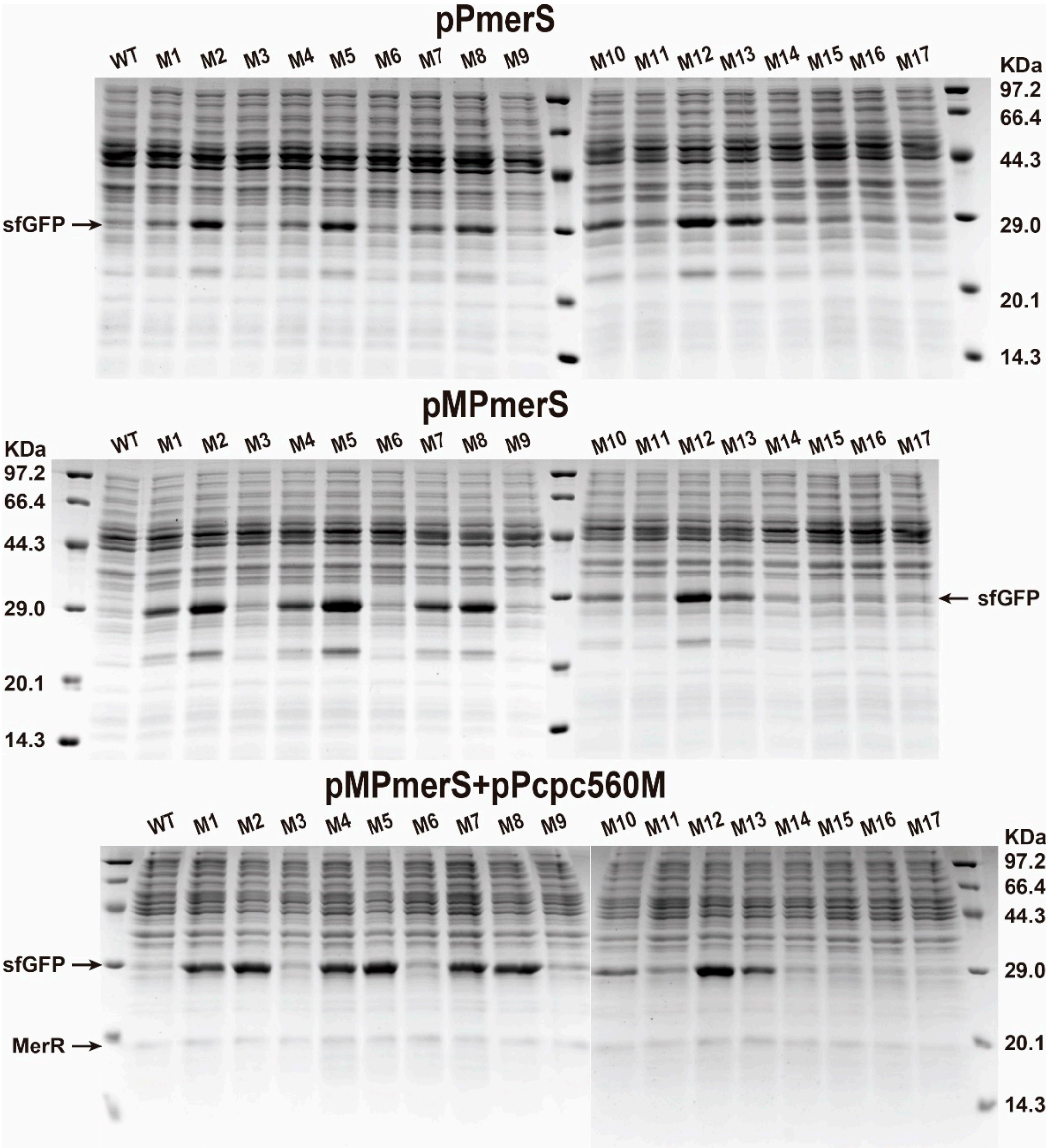

Fig.S7 The sfGFP expression levels from derivatives M1-M17 without mercury in three different systems (pPmerS, pMPmerS and pMPmerS+pPcpc560M).

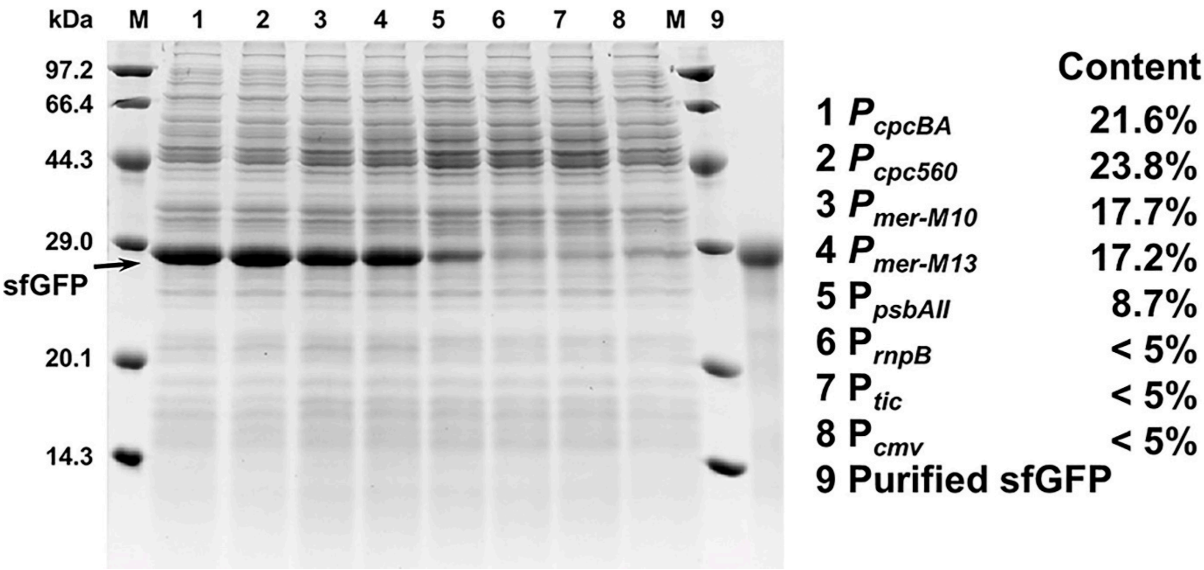

Fig.S8 Expression levels of sfGFP in *E. coli* from different promoters analyzed with Image Lab™ (Bio-Rad). Lanes 1 to 8 are promoters P_{cpcBA} (*cpcBA* promoter of *Synechocystis* sp. 6803, strong) [76], P_{cpc560} (*cpc560* promoter of *Synechocystis* sp. 6803, strong) [63], P_{mer}-M10 (*merT* promoter mutant M10 of *Pseudomonas aeruginosa,* strong), P_{mer}-M13 (*merT* promoter mutant M13 of *Pseudomonas aeruginosa,* strong), P_{psbAII} (*psbAII* promoter of *Synechocystis* sp. 6803) [64], P_{rnpB} (*rnpB* promoter of *Synechocystis* sp. 6803) [65], P_{tic} (*tic* promoter of *Synechocystis* sp. 6803) [66] and P_{cmv} (*cmv* promoter of Human betaherpesvirus) [67].

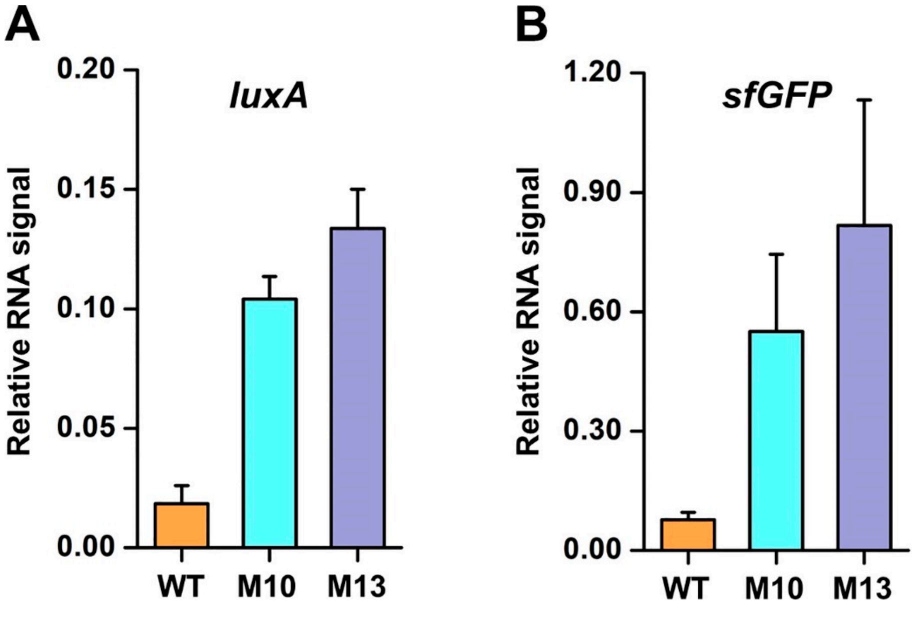

Fig.S9 Transcript levels of gene (*luxA* or *sfGFP*) downstream of the *mer* promoter (WT, derivatives M10 or M13) (A) RT-PCR assays for *luxA* gene. (B) RT-PCR assays for *sfGFP* gene. The control group was S16 rRNA. Transcript levels of the wild-type *mer* promoter are shown in orange, mutant M10 in cyan, and mutant M10 in slateblue.

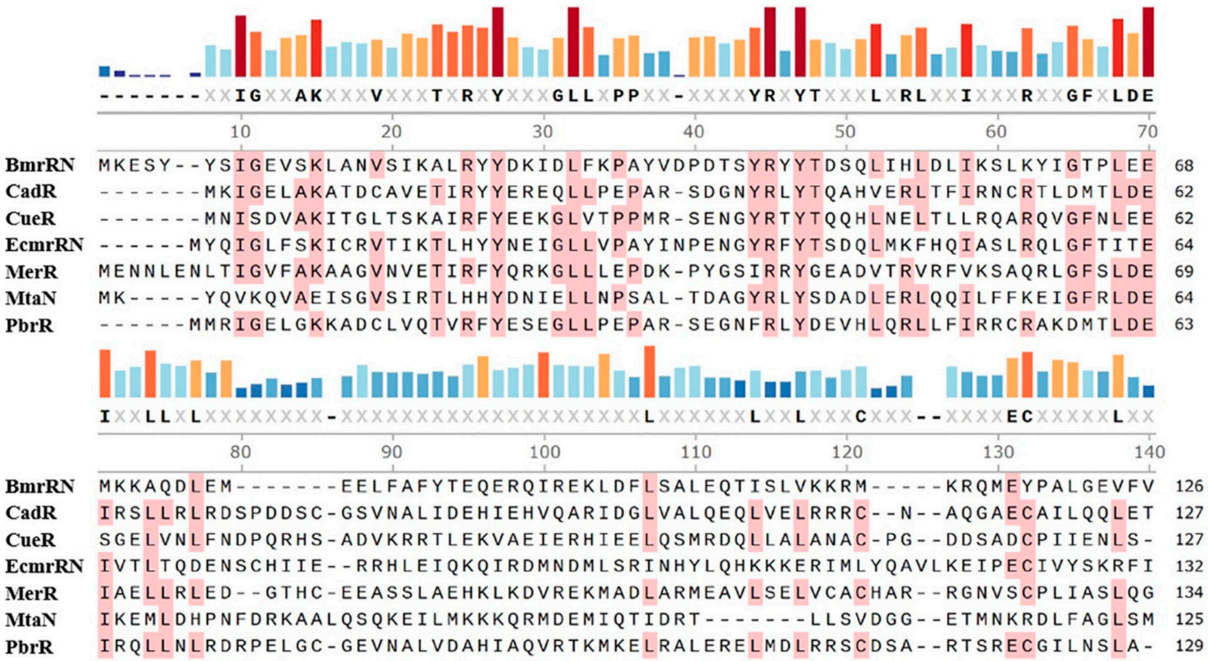

Fig.S10 Alignment of N-terminal sequences of MerR family transcription factors. BmrR (*Bacillus subtilis*, WP_003230325.1), CadR (*Pseudomonas putida*, WP_198743526.1), CueR (*Escherichia coli*, WP_089632711.1), EcmrR (*Escherichia coli*, WP_053276550.1), MerR (*Pseudomonas aeruginosa*, WP_003131969.1), MtaN (*Bacillus subtilis*, WP_038829835.1), PbrR (*Cupriavidus metallidurans*, WP_134593310.1).

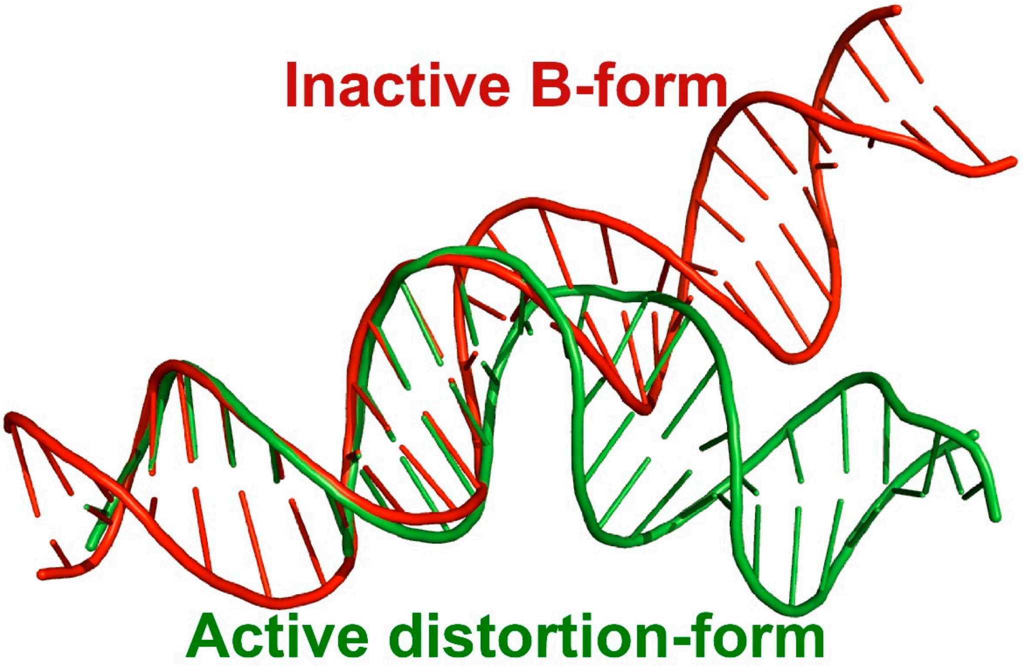

Fig.S11 Schematic comparison of the B-form promoter DNA and distorted promoter DNA. Non-activated B-form is red, activated distortion-form is green.

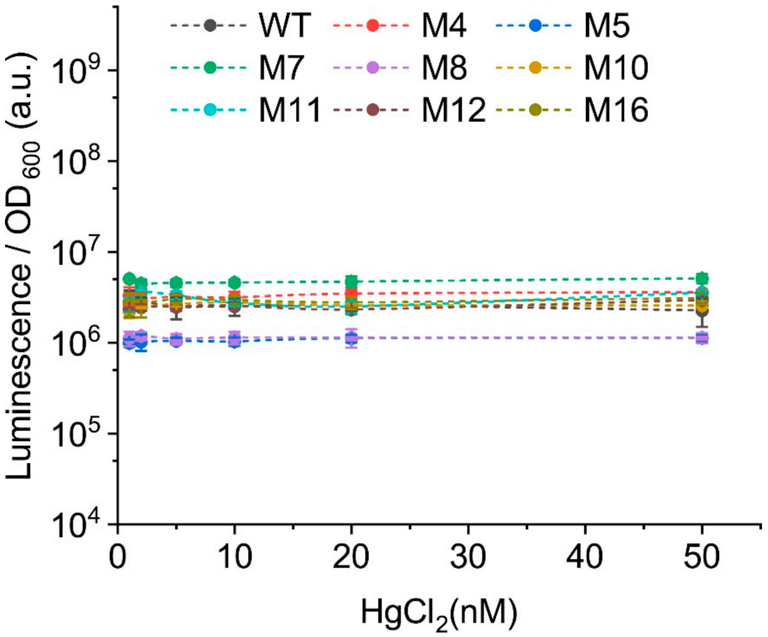

Fig.S12 Expression levels of the merR gene from wild type and different derivatives.

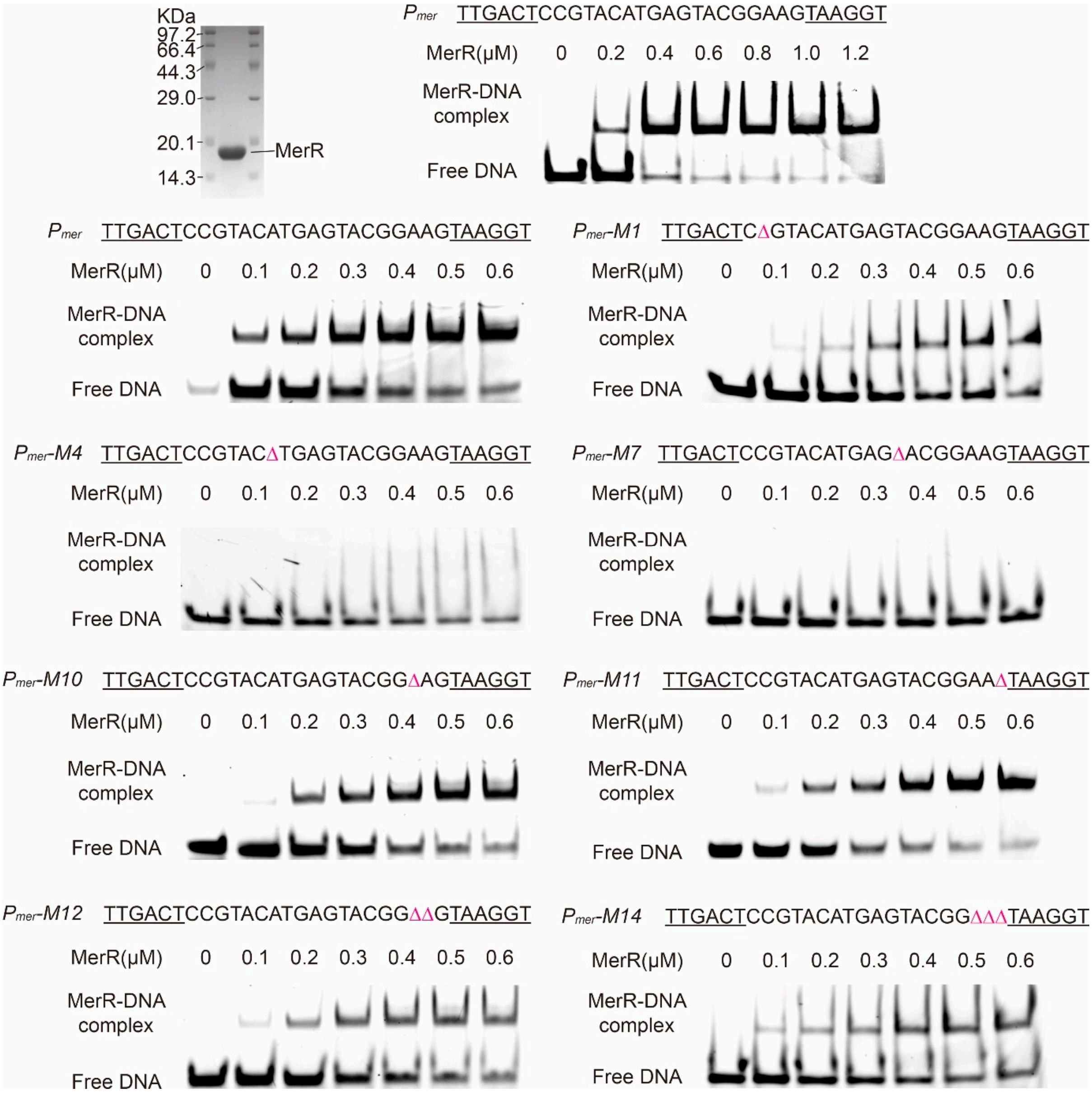

Fig.S13 MerR binding to promoter DNA of wild type and derivatives. Non-activated B-form is red, activated distortion-form is green. The promoter DNA fragments contain −35 to −10 elements and their spacer length ranges from 16-19 bp. 30 nM for DNA and 0, 100, 200, 300, 400, 500 and 600 nM for MerR were used in the final EMSA experiment.

BmrR ATCCGTTGACTCTCCCCTAGGAGGAGGTCTTACAGTATAAG
CadR GTGGCTTGACCCTATAGTGGCTACAGGGTGTTCACTTGGCA
CueR ATTTCTTGACCTTCCCCTTGCTGGAAGGTTTAACCTTTATC
EcmrR TGCCTTTGACCCTCCCCTAAGGGGAGGGTTTAGATTATCAG
MerR ATCGCTTGACTCCGTACATGAGTACGGAAGTAAGGTTACGC
MtaN GGGGATTGACCCTAACGTTGCGTGATTGTTTACGATAAAAA
PbrR ATGTCTTGACTCTATAGTAACTAGAGGGTGTTAAATCGGCA

Fig.S14 Sequence alignment of promoters with 19 bp spacer between −10 and −35 elements regulated by MerR family TFs. The −35 and −10 elements are marked with a double underline. Symmetrical sequences are shown in bold. BmrR (*Bacillus subtilis*, P_{bmr}, CP121266.1), CadR (*Pseudomonas putida*, P_{cad}, CP097525.1), CueR (*Escherichia coli*, P_{cue}, CP104618.1), EcmrR (*Escherichia coli*, P_{ecmr}) [43], MerR (*Pseudomonas aeruginosa*, P_{mer}, CP127126.1), MtaN (*Bacillus subtilis*, P_{mta}, CP127278.1), PbrR (*Cupriavidus metallidurans*, P_{pbr}, CP046332.1).

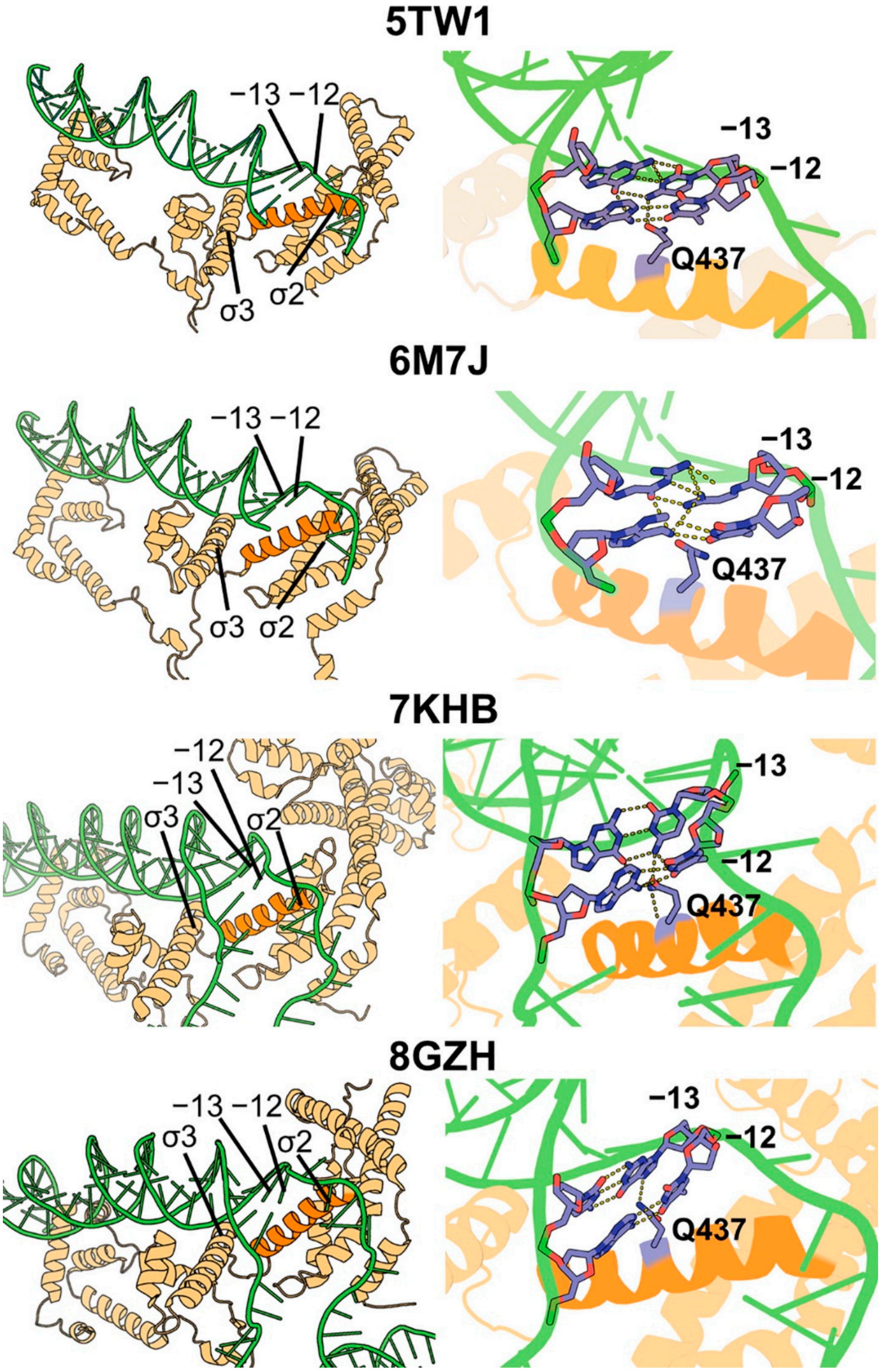

Fig.S15 Contact of region 2 (σ2) in sigma factor 70 (σ70) with promoter positions −13 and −12 in various RNAP holoenzyme complex crystals. The promoter DNA is green, the sigma factor is yellow and the σ2 is orange. The base pairs at positions −12 and −13 in the promoter and the

glutamine at position 437 (Q437) in σ2 are marked in purple. The yellow dotted lines show their interactions (hydrogen bonding). 5TW1 (PDB No.) is *Mtb* AP3 promoter from *Mycolicibacterium smegmatis* MC2 155 [72]. 6M7J (PDB No.) is *rrnA* P3 promoter and RNA polymerase from *Mycobacterium tuberculosis* [73]. 7KHB (PDB No.) is *rrnBP1* promoter and RNA polymerase from *Escherichia coli* K-12 [74]. 8GZH (PDB No.) is from *Synechocystis* sp. PCC 6803.

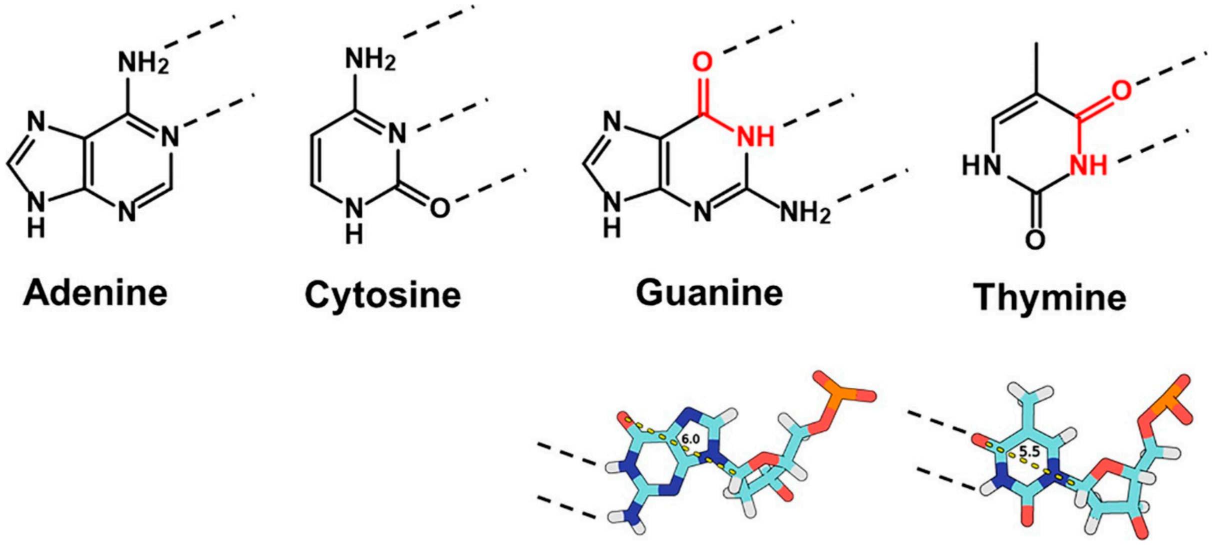

Fig.S16 Schematic structures of the four bases and common functional groups of guanine and thymine. Secondary amides, which form hydrogen bonds in guanine and thymine, are labelled in red.

Supporting Tables

Table S1 Promoter sequences.

Promoter	NCBI Genbank accession number	Sequences
P_{mer}	CP104985.1	ATCGCTTGACTCCGTACATGAGTACGGAAGTAAGGTTACGCTATCCAATTTCAATTCGAAAGGACAAGCGC
P_{cpcBA}	CP073017.1	GTTATAAAATAAACTTAACAAATCTATACCCACCTGTAGAGAAGAGTCCCTGAATATCAAAATGGTGGGATAAAAAGCTCAAAAAGGAAAGTAGGCTGTGGTTCCCTAGGCAACAGTCTTCCCTACCCCACTGGAAACTAAAAAAACGAGAAAAGTTCGCACCGAACATCAATTGCATAATTTTAGCCCTAAAACATAAGCTGAACGAAACTGGTTGTCTTCCCTTCCCAATCCAGGACAATCTGAGAATCCCCTGCAACATTACTTAACAAAAAAGCAGGAATAAAATTAACAAGATGTAACAGACATAAGTCCCATCACCGTTGTATAAAGTTAACTGTGGGATTGCAAAAGCATTCAAGCCTAGGCGCTGAGCTGTTTGAGCATCCCGGTGGCCCTTGTCGCTGCCTCCGTGTTTCTCCCTGGATTTATTTAGGTAATATCTCTCATAAATCCCCGGGTAGTTAACGAAAGTTAATGGAGATCAGTAACAATAACTCTAGGGTCATTACTTTGGACTCCCTCAGTTTATCCGGGGGAATTGTGTTTAAGAAAATCCCAACTCATAAAGTCAAGTAGGAGATTAATTCA
P_{cpc560}	CP073017.1	ACCTGTAGAGAAGAGTCCCTGAATATCAAAATGGTGGGATAAAAAGCTCAAAAAGGAAAGTAGGCTGTGGTTCCCTAGGCAACAGTCTTCCCTACCCCACTGGAAACTAAAAAAACGAGAAAAGTTCGCACCGAACATCAATTGCATAATTTTAGCCCTAAAACATAAGCTGAACGAAACTGGTTGTCTTCCCTTCCCAATCCAGGACAATCTGAGAATCCCCTGCAACATTACTTAACAAAAAAGCAGGAATAAAATTAACAAGATGTAACAGACATAAGTCCCATCACCGTTGTATAAAGTTAACTGTGGGATTGCAAAAGCATTCAAGCCTAGGCGCTGAGCTGTTTGAGCATCCCGGTGGCCCTTGTCGCTGCCTCCGTGTTTCTCCCTGGATTTATTTAGGTAATATCTCTCATAAATCCCCGGGTAGTTAACGAAAGTTAATGGAGATCAGTAACAATAACTCTAGGGTCATTACTTTGGACTCCCTCAGTTTATCCGGGGGAATTGTGTTTAAGAAAATCCCAACTCATAAAGTCAAGTAGGAGATTAATTCA
P_{psbAII}	CP073017.1	CTTTAGCGTTCCAGTGGATATTTGCTGGGGGTTAATGAAACATTGTGGCGGAACCCAGGGACAATGTGACCAAAAAATTCAGGGATATCAATAAGTATTAGGTATATGGATCATAATTGTATGCCCGACTATTGCTTAAACTGACTGACCACTGACCTTAAGAGTAATGGCGTGCAAGGCCCAGTGATCAATTTCATTATTTTTCATTATTTCATCTCCATTGTCCCTGAAAATCAGTTGTGTCGCCCCTCTACACAGCCCAGAACTATGGTAAAGGCGCACGAAAAACCGCCAGGTAAACTCTTCTCAACCCCCAAAACGCCCTCTGTTTACCCATGGAAAAAACGACAATTACAAGAAAGTAAAACTTATGTCATCTATAAGCTTCGTGTATATTAACTTCCTGTTACAAAGCTTTACAAAACTCTCATTAATCCTTTAGACTAAGTTTAGTCAGTTCCAATCT

		GAACATCGACAAATACATCATAAGGAATTAT
P_{rnpB}	CP073017.1	TTCAATGCGGTCCAATACCTCCCCTGCCCAACTGGGTAAGCTCGCG GCTCCACTGAGTAATACAGACAAGGCTAAACAGGCAAATTTTTCA TTGGTCAACTCCTAGCACCAATTTCCCAAGACTACGGAGGGGGCA ATGAAGTTTCAATTAATTGGGGTCACAAACCACAGCGGCCTATGGC TCTAATCAATGGCACACTAGAAAAATAGTGGAGGT
P_{tic}	KM984770.1	TTGACAATTAATCATCGCGGCTCGTATAATGTGTGGTCACACAGGA AACAGAAT
P_{cmv}	KU550088.1	CGTTACATAACTTACGGTAAATGGCCCGCCTGGCTGACCGCCCAAC GACCCCCGCCCATTGACGTCAATAATGACGTATGTTCCCATAGTAAC GCCAATAGGGACTTTCCATTGACGTCAATGGGTGGAGTATTTACGG TAAACTGCCCACTTGGCAGTACATCAAGTGTATCATATGCCAAGTAC GCCCCCTATTGACGTCAATGACGGTAAATGGCCCGCCTGGCATTATG CCCAGTACATGACCTTATGGGACTTTCCTACTTGGCAGTACATCTAC GTATTAGTCATCGCTATTACCATGGTGATGCGGTTTTGGCAGTACAT CAATGGGCGTGGATAGCGGTTTGACTCACGGGGATTTCCAAGTCTC CACCCCATTGACGTCAATGGGAGTTTGTTTTGGCACCAAAATCAAC GGGACTTTCCAAAATGTCGTAACAACTCCGCCCCATTGACGCAAAT GGGCGGTAGGCGTGTACGGTGGGAGGTCTATATAAGCAGAGCT

Table S2 Primers for the template plasmid construction.

Name	Sequences
merR F	GATCTCTAGACTACGGCATAGCAGAACCAGC
P_{mer}-*luxAB* R	ATGTAAGCAAAAAGTTTCCAAATTTCATGCGCTTGTCCTTTCGAAT
P_{mer}-*luxAB* F	ATTCGAAAGGACAAGCGCATGAAATTTGGAAACTTTTTGCTTACAT
luxAB R	GTACGAATTCTTAGGTATATTCCATGTGGTACTTCTT
P_{mer}-*sfGFP* R	AGTTCTTCGCCTTTGCTCATGCGCTTGTCCTTTCGAAT
P_{mer}-*sfGFP* F	ATTCGAAAGGACAAGCGCATGAGCAAAGGCGAAGAACT
sfGFP R	GTACGAATTCTTACTTGTACAGCTCGTCCATG
P_{mer} F	GATCTCTAGAATCGCTTGACTCCGTACATG
luxAB F	GATCTCTAGATTAGGTATATTCCATGTGGTACTTCTTAATATTATCA
luxAB-P_{mer} R	GTCAAGCGATATGAAATTTGGAAACTTTTTGCTTACATACC
luxAB-P_{mer} F	CAAAAAGTTTCCAAATTTCATATCGCTTGACTCCGTACATG
P_{cpc560} *F*	GTACGAGCTCACCTGTAGAGAAGAGTCCCTGA
P_{cpc560}-*merR* R	GTAGGAGATTAATTCAATGGAAAATAACCTGGAAAACCTGAC
P_{cpc560}-*merR* F	CCAGGTTATTTTCCATTGAATTAATCTCCTACTTGACTTTATGAGTTGG
merR R	GATCCTCGAGCTACGGCATAGCAGAACCAGC
P_{cpcBA}-*sfGFP F*	GATCTCTAGAGTTATAAAATAAACTTAACAAATCTATACCCACCTGT
P_{cpc560}-*sfGFP F*	GATCTCTAGAACCTGTAGAGAAGAGTCCCTG
P_{psbAII}-*sfGFP F*	GATCTCTAGACTTTAGCGTTCCAGTGGATATTTGC
P_{tic}-*sfGFP F*	GATCTCTAGATTGACAATTAATCATCGCGGCTC
P_{cmv}-*sfGFP F*	GATCTCTAGACGTTACATAACTTACGGTAAATGGCC

Table S3 Mutagenic primers for the *mer* promoter.

Name	Sequences
Del −31/−30 F	AGGTTATTTTCCATATCGCTTGACTCGTACATGAGTACGGAAGTAAGGTTACG
Del −31/−30 R	CGTAACCTTACTTCCGTACTCATGTACGAGTCAAGCGATATGGAAAATAACCT
Del −30to−29 F	AGGTTATTTTCCATATCGCTTGACTCTACATGAGTACGGAAGTAAGGTTACG
Del −30to−29 R	CGTAACCTTACTTCCGTACTCATGTAGAGTCAAGCGATATGGAAAATAACCT
Del −30to−28 F	AGGTTATTTTCCATATCGCTTGACTCACATGAGTACGGAAGTAAGGTTACG
Del −30to−28 R	CGTAACCTTACTTCCGTACTCATGTGAGTCAAGCGATATGGAAAATAACCT
Del −25 F	ATCGCTTGACTCCGTACTGAGTACGGAAGTAAGG
Del −25 R	CCTTACTTCCGTACTCAGTACGGAGTCAAGCGAT
Del −25to−24 F	TCGCTTGACTCCGTACGAGTACGGAAGTAAGG
Del −25to−24 R	CCTTACTTCCGTACTCGTACGGAGTCAAGCGA
Del −25to−23 F	ATCGCTTGACTCCGTACAGTACGGAAGTAAGGTT
Del −25to−23 R	AACCTTACTTCCGTACTGTACGGAGTCAAGCGAT
Del −20 F	TTGACTCCGTACATGAGACGGAAGTAAGGTTACG
Del −20 R	CGTAACCTTACTTCCGTCTCATGTACGGAGTCAA
Del −19to−20 F	TTGACTCCGTACATGAGCGGAAGTAAGGTTACGC
Del −19to−20 R	GCGTAACCTTACTTCCGCTCATGTACGGAGTCAA
Del −18to−20 F	TTGACTCCGTACATGAGGGAAGTAAGGTTACGCT
Del −18to−20 R	AGCGTAACCTTACTTCCCTCATGTACGGAGTCAA
Del −15/−14 F	GACTCCGTACATGAGTACGGAGTAAGGTTACGCTATCCAATTTCA
Del −15/−14 R	TGAAATTGGATAGCGTAACCTTACTCCGTACTCATGTACGGAGTC
Del −13 F	CCGTACATGAGTACGGAATAAGGTTACGCTATCCAA
Del −13 R	TTGGATAGCGTAACCTTATTCCGTACTCATGTACGG
Del −15to−14 F	TCCGTACATGAGTACGGGTAAGGTTACGCTATCC
Del −15to−14 R	GGATAGCGTAACCTTACCCGTACTCATGTACGGA
Del −13to−14 F	CCGTACATGAGTACGGATAAGGTTACGCTATCCA
Del −13to−14 R	TGGATAGCGTAACCTTATCCGTACTCATGTACGG
Del −13to−15 F	TCCGTACATGAGTACGGTAAGGTTACGCTATCCA
Del −13to−15 R	TGGATAGCGTAACCTTACCGTACTCATGTACGGA
G−13A F	CCGTACATGAGTACGGAAATAAGGTTACGCTATCC
G−13A R	GGATAGCGTAACCTTATTTCCGTACTCATGTACGG
G−13T F	CCGTACATGAGTACGGAATTAAGGTTACGCTATCC
G−13T R	GGATAGCGTAACCTTAATTCCGTACTCATGTACGG
G−13C F	CCGTACATGAGTACGGAACTAAGGTTACGCTATCC
G−13C R	GGATAGCGTAACCTTAGTTCCGTACTCATGTACGG

Table S4 DNA for the electrophoretic mobility shift assay (EMSA).

Name	Sequences	Length
P_{mer} F	TTGACTCCGTACATGAGTACGGAAGTAAGGT	31 bp
P_{mer} R	ACCTTACTTCCGTACTCATGTACGGAGTCAA	31 bp
P_{mer}-M1 F	TTGACTCGTACATGAGTACGGAAGTAAGGT	30 bp
P_{mer}-M1 R	ACCTTACTTCCGTACTCATGTACGAGTCAA	30 bp
P_{mer}-M4 F	TTGACTCCGTACTGAGTACGGAAGTAAGGT	30 bp
P_{mer}-M4 R	ACCTTACTTCCGTACTCAGTACGGAGTCAA	30 bp
P_{mer}-M7 F	TTGACTCCGTACATGAGACGGAAGTAAGGT	30 bp
P_{mer}-M7 R	ACCTTACTTCCGTCTCATGTACGGAGTCAA	30 bp
P_{mer}-M10 F	TTGACTCCGTACATGAGTACGGAGTAAGGT	30 bp
P_{mer}-M10 R	ACCTTACTCCGTACTCATGTACGGAGTCAA	30 bp
P_{mer}-M11 F	TTGACTCCGTACATGAGTACGGAATAAGGT	30 bp
P_{mer}-M11 R	ACCTTATTCCGTACTCATGTACGGAGTCAA	30 bp
P_{mer}-M12 F	TTGACTCCGTACATGAGTACGGGTAAGGT	29 bp
P_{mer}-M12 R	ACCTTACCCGTACTCATGTACGGAGTCAA	29 bp
P_{mer}-M14 F	TTGACTCCGTACATGAGTACGGTAAGGT	28 bp
P_{mer}-M14 R	ACCTTACCGTACTCATGTACGGAGTCAA	28 bp

The −35 and −10 elements are marked with an underscore.

Table S5 Expression activity of *mer* promoter derivatives in the pMPmerS plasmid.

NO.	Promoter	Expression level (sfGFP)
		pMPmerS (Without Hg(II))
1	$P_{mer\text{-}M1}$	777
2	$P_{mer\text{-}M2}$	1306
3	$P_{mer\text{-}M3}$	56
4	$P_{mer\text{-}M4}$	759
5	$P_{mer\text{-}M5}$	1551
6	$P_{mer\text{-}M6}$	74
7	$P_{mer\text{-}M7}$	869
8	$P_{mer\text{-}M8}$	1251
9	$P_{mer\text{-}M9}$	96
10	$P_{mer\text{-}M10}$	511
11	$P_{mer\text{-}M11}$	132
12	$P_{mer\text{-}M12}$	1074
13	$P_{mer\text{-}M13}$	568
14	$P_{mer\text{-}M14}$	179

Table S6 Primers for Reverse-transcription PCR (RT-PCR) assay.

Name	Sequences
16S rRNA F	CGTGTATGAAGAAGGCCTTCG
16S rRNA R	CTGAGCGTCAGTCTTCGTCC
sfGFP F	CTGCTGCCGGACAATCACTA

sfGFP R	ATGCCATGAGTGATACCCGC
luxA F	GTGCCGAATACGCGAAAGTC
luxA R	CCCATCCAGTACGCCAACTT
